# Transcriptomic profiling of peripheral blood cells in HPV‐associated carcinoma patients receiving combined valproic acid and avelumab

**DOI:** 10.1002/1878-0261.13519

**Published:** 2023-09-17

**Authors:** Najmeh Bozorgmehr, Hussain Syed, Siavash Mashhouri, John Walker, Shokrollah Elahi

**Affiliations:** ^1^ Division of Foundational Sciences, School of Dentistry University of Alberta Edmonton AB Canada; ^2^ Department of Medical Oncology University of Alberta Edmonton AB Canada; ^3^ Faculty of Medicine and Dentistry Li Ka Shing Institute of Virology University of Alberta Edmonton AB Canada

**Keywords:** IL‐18, IL‐8, immunotherapy, prognostic biomarker, RNA sequencing

## Abstract

Human papillomavirus (HPV)‐associated cancer continues to evade the immune system by promoting a suppressive tumor microenvironment. Therefore, immunotherapy appears to be a promising approach for targeting HPV‐associated tumors. We hypothesized that valproic acid (VA) as an epigenetic agent combined with avelumab may enhance the antitumor immunity in HPV‐associated solid tumors. We performed bulk RNA‐sequencing (RNA‐Seq) on total peripheral blood mononuclear cells (PBMCs) of seven nonresponders (NRs) and four responders (Rs). A total of 39 samples (e.g., pretreatment, post‐VA, postavelumab, and endpoint) were analyzed. Also, we quantified plasma analytes and performed flow cytometry. We observed a differential pattern in immune response following treatment with VA and/or avelumab in NRs vs. Rs. A significant upregulation of transcripts associated with NETosis [the formation of neutrophil extracellular traps (NETs)] and neutrophil degranulation pathways was linked to the presence of a myeloid‐derived suppressor cell signature in NRs. We noted the elevation of IL‐8/IL‐18 cytokines and a distinct transcriptome signature at the baseline and endpoint in NRs. By using the receiver operator characteristics, we identified a cutoff value for the plasma IL‐8/IL‐18 to discriminate NRs from Rs. We found differential therapeutic effects for VA and avelumab in NRs vs. Rs. Thus, our results imply that measuring the plasma IL‐8/IL‐18 and bulk RNA‐Seq of PBMCs may serve as valuable biomarkers to predict immunotherapy outcomes.

## Graphical abstract

Nonresponders to immunotherapy showed higher plasma IL‐8, IL‐18, and VEGF levels, along with increased myeloid cell/NET formation but decreased CD8^+^ T cells. Responders had lower plasma IL‐8, IL‐18, fewer myeloid cells, but higher CD8^+^ T‐cell proportions.

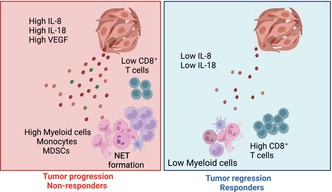

**Abbreviation**
CECs: CD71^+^ erythroid cells
CTLA‐4: cytotoxic T lymphocyte‐associated protein 4
DC: dendritic cells
dsDNA: double‐stranded DNA virus
EBV: Epstein–Barr virus
EDTA: ethylenediamineteraacetic acid
EP: endpoint
FCRL6: FC receptor‐like
HBEFF: heparin‐binding EGF‐like factor
HDAC: histone deacetylase
HPV: human papillomavirus
HRE: hypoxia‐response element
iCCR: confirmed complete response
ICIs: immune checkpoint inhibitors
iCPD: immune confirmed progressive disease
iCPR: confirmed partial response
iSD: stable disease
iUPDs: immune unconfirmed progressive disease
JAK/STAT: Janus‐kinase/signal transducer and activator of transcription
LAIR‐1: leukocyte‐associated immunoglobulin‐like receptor 1
MCP‐4: monocyte chemoattractant protein 1
MDC: macrophage‐derived chemokine
MDSCs: myeloid‐derived suppressor cells
MG‐CSF: granulocyte‐monocyte colony‐stimulating factor
MIP‐1: macrophage inflammatory protein 1
NETs: neutrophil extracellular traps
NRs: nonresponders
PBMCs: peripheral blood mononuclear cells
PD‐1: programmed death 1
PD‐L1: programmed death‐ligand 1
PMN: polymorphonuclear
RLD: regularized logarithmic‐transformed data
RNA‐Seq: RNA‐sequencing
Rs: responders
TAMs: tumor‐associated macrophages
TARC: thymus‐and‐activation‐regulated chemokine
THBS: thrombospondin‐1
TLRs: Toll‐like receptors
TME: tumor microenvironment
Tregs: regulatory T cells
VASTs: virus‐associated solid tumors
VP: valproic acid
VSGF: vascular endothelial growth factor

## 1 Introduction

Tumor cells exploit diverse mechanisms to suppress or evade immune surveillance to facilitate tumor progression and metastasis [1]. One mechanism is related to the recruitment and education of different suppressor cells to build an immunosuppressive tumor microenvironment (TME), consisting of myeloid‐derived suppressor cells (MDSCs), neutrophils, tumor‐associated macrophages (TAMs), regulatory T cells (Tregs), and CD71^+^ erythroid cells (CECs) [2, 3, 4, 5]. Moreover, tumor‐associated soluble mediators (e.g., cytokines), altered metabolism, aberrant vessels, and curbed stromal cells can influence the immune response against the tumor [6]. Although immune checkpoint inhibitors (ICIs) targeting PD‐1, PD‐L1, and CTLA‐4 have revolutionized the cancer field, only some patients exhibit durable clinical outcomes [7]. Other patients either do not respond (nonresponders (NRs) or initially respond but eventually acquire resistance [8, 9]. Hence, this remains a prevailing clinical need in the ICI field to discriminate NRs from responders (Rs) at the baseline or during treatment. Such studies to identify potential Rs from NRs will save lives and facilitate clinical decision‐making.

There is growing evidence that tumor recruits and manipulates myeloid cells to transform them into immunosuppressive cells as an antitumor immune response evasion mechanism [10, 11]. Myeloid cells constitute the majority of peripheral blood immune cells and include different cell subsets with distinct functional properties [1]. Granulocytes, monocytes, and dendritic cells are the major innate immune components in the periphery. The TME of solid tumors is a fertile niche to recruit and educate MDSCs, divided into monocytic (M‐MDSC) and granulocytic (PMN‐MDSCs) suppressor cells [12]. Given their immunosuppressive nature, it is evident that these myeloid cells suppress T‐cell effector functions and interfere with the efficacy of ICIs [13]. Accordingly, targeting myeloid cells to improve the clinical outcomes of ICIs has been considered [14]. Although ICIs in solid tumors have been widely investigated, their application in virus‐associated tumors merits further investigation.

Human papilloma virus (HPV) is dsDNA virus. Several high‐risk HPV genotypes (e.g., HPV‐16, ‐18) integrate into the host's DNA and cause squamous cell carcinoma of the cervix, anus, vulva, penis, and oropharynx [15]. Generally, HPV‐associated carcinomas are genetically less complex and induce a potent antitumoral response leading to improved overall survival [15]. Nevertheless, often the diagnosis of these tumors occurs very late when tumors are locally advanced or become metastatic [16]. Immunotherapy appears to be a promising approach for treating HPV‐ associated tumors due to the complex virus and host interactions [17]. Mounting evidence has shown that HPV‐associated carcinomas are potential candidates for ICIs since they modulate the expression of co‐inhibitory receptors [18, 19, 20]. For example, the E7 oncoprotein generated by HPV‐16 augments PD‐L1 expression [21], and similarly, E6 and E7 oncoproteins induce hypermethylation of DNA repair genes and prompt CTLA‐4 upregulation [22]. Alongside FDA has approved pembrolizumab (anti‐PD‐1) therapy for recurrent or metastatic cervical cancer [23]. However, failure to respond to such treatments has raised the necessity of combined treatment options. For instance, ICIs combined with histone deacetylases (HDACs) inhibitors have shown promising results by enhancing antitumor immunity [24].

Valproic acid is an HDAC (Histone deacetylase) inhibitor and is a common medication for treating neurologic disorders, but its promising therapeutic property against HIV and cancer has already been reported [25]. Genetic modifications in DNA are linked to cancer progression, and HDACs are essential regulators of gene expression [26]. Given HDACs' dysregulation in different cancers, they could be potential therapeutic targets. Almost all cancer cells exhibit increased HDAC activity that impacts gene expression and cell differentiation in different aspects [27]. These gene transcriptional changes impact DNA repair, cell cycle control, apoptosis, differentiation, nuclear import, metabolism, and vascular function [25]. VA is widely studied in different cancers due to its apoptotic effects on malignant cells [28, 29]. Reports have shown that VA halts the proliferation of HPV‐associated cancer cell lines [30] and increases cytotoxic T lymphocyte response to HPV‐associated cervical cancers [31]. Also, it is reported that VA has various immunological properties and modulates both innate and adaptive immune responses [32].

One of the significant challenges in the ICI field is potential predictive biomarkers to identify patients who will benefit or respond positively to ICIs. Due to the complexity of genetic host factors, tumor types, tumor mutations, and differential immune responses following ICI treatment [9], identifying potentially predictive biomarkers is highly valuable. In this study, we utilized bulk RNA sequencing (RNA‐Seq) of PBMCs from HPV‐associated carcinoma patients to identify Rs from NRs. Patients were enrolled in a nonrandomized, single‐arm, phase II clinical trial investigating the effects of oral valproate combined with avelumab (anti‐PD‐L1) [33]. Given the impracticality of tumor tissue biopsies, our results support the value of bulk RNA‐Seq of the peripheral blood as a noninvasive approach to predict potential response to ICIs. We anticipate our results will improve clinical decision‐making in ICIs and inform further investigations.

## 2 Materials and methods

### 2.1 Study population

This study was a nonrandomized, single‐arm, basket phase II clinical trial investigating the effects of oral valproate combined with avelumab (anti‐PD‐L1) in patients with virus‐associated solid tumors (VASTs) [33, 34]. Peripheral blood samples were collected in EDTA tubes from advanced HPV‐associated carcinoma (*n* = 11 out of 40 patients) who enrolled in the LATENT trial study (Lytic Activation To Enhance Neoantigen‐directed Therapy) at the Cross Cancer Institute, University of Alberta between January 2018 and March 2020. All tumors were p16‐positive squamous cell carcinoma from different sites (e.g., genitourinary, anal, head, and neck). Clinical data and immune response to treatment based on iRECIST criteria [35] are described in Table 1.

**Table 1:** Patients' characteristics. F, female; iCCR, confirmed complete response; iCPD, confirmed progressive disease; iCPR, confirmed partial response; iSD, stable disease; iUPD, unconfirmed progressive disease; iUPR, unconfirmed partial response; M, male; NE, response was not evaluated; NR, nonresponders (iCPD^+^ iUPD^+^ Clinically progressive); R, responders (iCCR^+^ iCPR^+^iUPR^+^ iSD).

Response category	Sex	Primary tumor site	Stage	Best response
R1	F	Anal	IV	iCCR
R2	F	Unknown	IV	iCPR
R3	M	Tonsil	II	iSD
R4	F	Cervix	IV	iSD
NR1	F	Cervix	III	iCPD
NR2	M	Oropharynx	IVB	iCPD
NR3	M	Tonsil	III	NE/clinically progressive
NR4	F	Vulva	III	iUPD
NR5	F	Anal	IV	iUPD
NR6	F	Anal	IV	iUPD
NR7	M	Anal	IV	iUPD

### 2.2 Ethics statement

This study was approved by the Health Research Ethics Board of Alberta (HREBA protocol # HREBA CC‐17‐0374) at the University of Calgary and conducted to the standards set by the Declaration of Helsinki. Written informed consent to participate in the study was obtained from all participants.

### 2.3 Cell isolation

Peripheral blood mononuclear cells (PBMCs) were isolated from fresh blood using Ficoll‐Paque gradients (GE Healthcare, Chicago, IL, USA). RPMI 1640 (Sigma‐Aldrich, Burlington, MA, USA) supplemented with 10% FBS (Sigma‐Aldrich), and 1% penicillin/streptomycin (Sigma‐Aldrich) was used for PBMC culture. Isolated PBMCs were cryopreserved in TRIzol (Invitrogen, Waltham, MA, USA) for further RNA isolation.

### 2.4 Library construction and sequencing

Total RNA (39 samples from 11 patients) was extracted from cryopreserved PBMCs in TRIzol reagent (Direct‐zol RNA kit) based on the manufacturer's protocol, as reported elsewhere [36]. Samples' characteristics are summarized in Table S1. Isolated RNA was quantified, and the quality of RNA samples was assessed by Agilent Bioanalyzer. Samples with RNA integrity numbers (RIN) of more than 6.5 were selected and purified (Fig. S1A and Table S1) using Poly A selection with oligo dTs conjugated to paramagnetic beads. As per the kit's instruction, the first and second strands of cDNA libraries were constructed from 100 ng of extracted RNA using the TruSeq RNA Library Prep kit v2 (Illumina). T‐A ligation was added to the blunted and A‐tailed cDNA before doing 12 cycles of PCR to incorporate illumine adapters containing multiplexing barcodes. A HiSeq 2500 Illumina instrument sequenced samples on a paired‐end 150 cycles protocol. Demultiplexed data generated are available from the SRA portal on NCBI under accession number GSE229014.

### 2.5 Bioinformatic analyses

kallisto software (KALLISTO software GmbH, Hoxter, Germany) aligned fragments to the human cDNA database (GRCh38). One hundred permutations were selected during pseudo‐alignments and bias corrections. For statistical analysis, patients were divided into two groups Rs (*n* = 4) and NRs (*n* = 7). Statistics was done using r package version 4.2.0 (2022 The R Foundation for Statistical Computing Platform: x86_64‐apple‐darwin17.0). Differential expression analyses of count data were done by DESeq2 method [37] as calculated shrinkage estimation for dispersions and fold changes. The count data were converted into matrix data sets as below: Cross‐sectional group comparisons (between R and NR at baseline and endpoint) were designed as per group, and longitudinal (R at cycles 1, 2, and endpoint vs. cycle 0, NR at cycles 1, 2, and endpoint vs. cycle 0) that matrix data sets were designed as per patient group. Data were normalized using RLD (regularized logarithmic‐transformed data). A list of differentially expressed transcripts was obtained for each pairwise comparisons: Abundance differences were considered as differentially expressed with an absolute Log_2_ fold change > 1 and *P*adj value < 0.05 (*False Discovery Rate (FDR)* < 0.05). Euclidian distances between samples were calculated by RLD and visualized using pheatmap package [38], which was used for heatmap plots and ggplot2 package (v3. 3.3) [39, 40] for PCA (Principal component Analysis) and volcano plots. ipa software (QIAGEN Inc., https://www.qiagenbioinformatics.com/products/ingenuity‐pathwayanalysis; 2023) [41] was used for enrichment and functional analysis. As proof, results were also compared with metascape (v3.5.20230101) [42] software.

### 2.6 Digital cytometry

The CIBERSORTx from Newman Lab (https://cibersortx.stanford.edu/) [43] is used to deconvolute immune cell fractions and abundances from the isolated PBMCs' bulk RNA‐Seq data [44]. The TPM (Transcripts per million) files, representative of the gene length‐normalized expression data, were uploaded as the mixture of gene files to the CIBERSORTx website and compared with software incorporated LM22 signature matrix file. The type and abundance of immune cells were merged into six cell types, including B cells, CD8^+^ T cells, CD4^+^ T cells, natural killer (NK) cells, monocytes, and granulocytes. The *P*‐values < 0.05 were considered statistically significant.

### 2.7 Flow cytometry

Conjugated fluorophore monoclonal antibodies were purchased from BD Biosciences, Ashland, OR, USA, Thermo Fisher Scientific, Waltham, MA, USA, and BioLegend, San Diego, CA, USA. The list of antibodies used for this study is as follows: anti‐CD3 (SK7), anti‐CD4 (RPA‐T4), anti‐CD8 (RPA‐T8), CD71 (MA712), CD235a (HIR2), anti‐CD14 (M5E2), and anti‐CD11b (ICRF44). As reported previously, surface staining was carried out *ex vivo* using fresh PBMCs [33, 45]. Live/dead staining (Thermo Fisher Scientific) was performed to exclude dead cells. LSR‐Fortessa SORP flow cytometry machine and flow jo software (V.10.8.1; BD Biosciences) were used for data acquirement and analysis.

### 2.8 ELISA and Multiplex assays

Multiplex analyses of plasma cytokines/chemokines were conducted by Mesoscale Discovery (MSD) kits, as we have reported before [45, 46]. Data were obtained on a V‐plex® Sector Imager 2400 plate reader and analyzed on the msd workbench 3.0 software (MSD, Rockville, MD, USA). Moreover, plasma soluble Galectin‐9, PD‐L1, IL‐18, and TGF‐β were detected by DuoSet ELISA kit (R&D systems, Inc., Minneapolis, MN, USA) following the manufacturer's protocols. Synergy H1 Biotek microplate reader was used to acquire the ELISA data and analyzed by gen5 V.2.07 software. Also, plasma samples from 10 patients (Table S2) enrolled in this clinical trial were used to validate soluble mediator measurement as an ancillary cohort.

### 2.9 Statistical analysis

We used graph pad prism software (Version 9.5.0; GraphPad Software Boston, MA, USA) for the statistical analysis. The Mann–Whitney *U* or Wilcoxon signed‐rank test was used appropriately for nonpaired or paired comparisons. *P*‐values < 0.05 were counted as statistically significant. Data showed as Mean ± SEM.

## 3 Results

### 3.1 Clinical trial outlines and patient characteristics

**Fig. 1:**
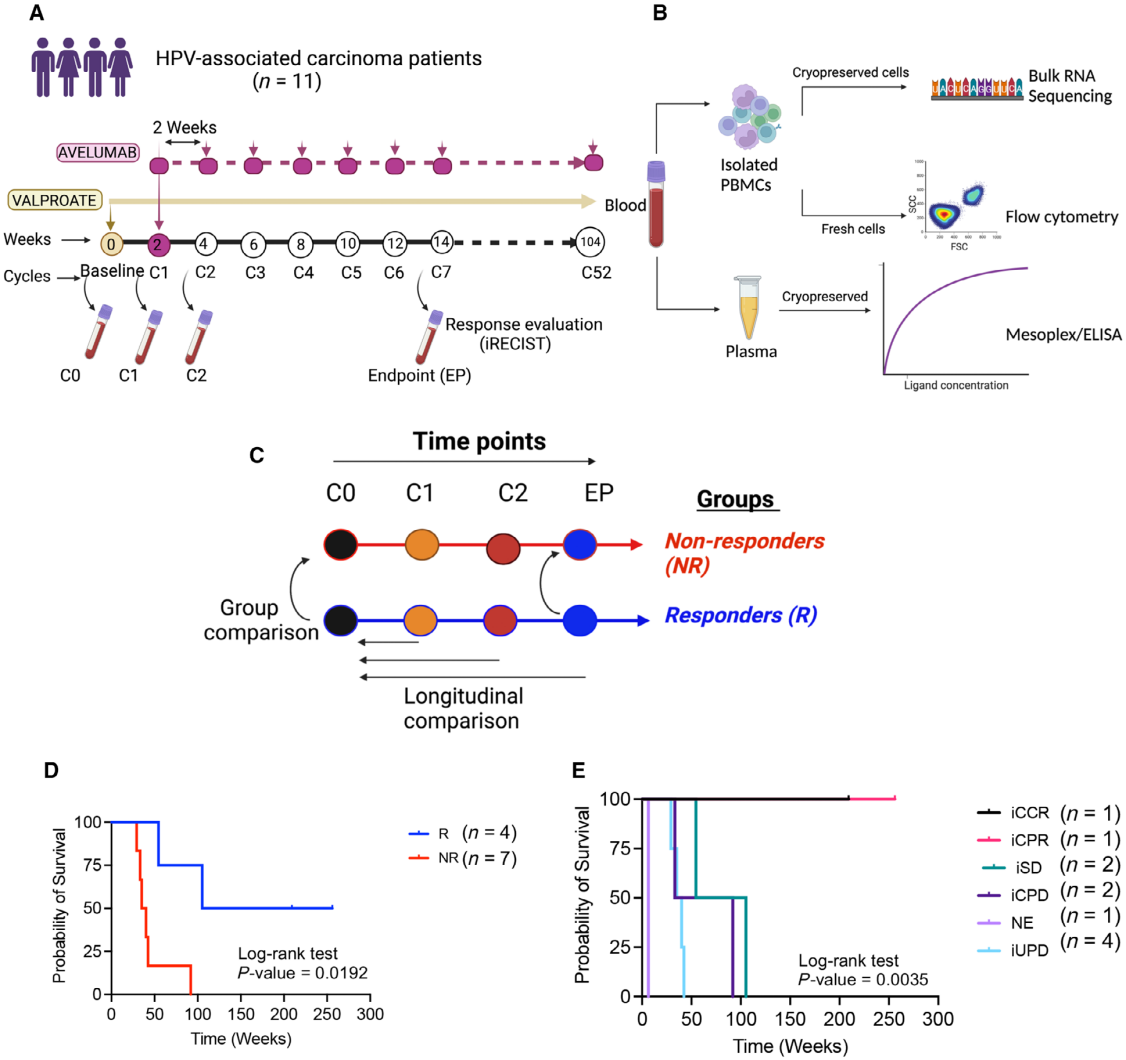
Overview of the clinical trial and experimental design. (A) Metastatic HPV‐associated carcinoma patients received daily valproic acid (VA) at the initiation of the trial or cycle 0 for 2 weeks, from Week 2 (cycle 1), they received avelumab (anti‐PD‐L1) every 2 weeks combined with daily VA throughout the study. At the baseline (Cycle 0 = C0), cycle 1 (C1), cycle 2 (C2), and cycle 6/7 (Endpoint = EP), blood was collected. (B) Peripheral blood mononuclear cells (PBMCs) were isolated for bulk RNA sequencing (RNA‐Seq) and flowcytometry, plasma was separated for Mesoplex/ELISA study. (C) Analysis flowchart showing group comparison between responders (Rs), nonresponders (NRs), and comparing time points in each group to the baseline. (D) The survival probability in Rs vs. NRs up to 250 weeks. (E) The survival probability of different patients based on iRECIST criteria.

The LATENT Clinical Trial study (ClinicalTrials.gov Identifier: NCT03357757) [34] was an open‐label, nonrandomized, single‐arm phase II to investigate the effects of combined valproic acid and avelumab (anti‐PD‐L1) in a cohort of 40 virus‐associated solid tumors (https://classic.clinicaltrials.gov/ct2/show/NCT03357757). The treatment started with oral daily VA followed by avelumab (10 mg/kg, i.v., every 2 weeks) (Fig. 1A). Every 2 weeks counted as a cycle (C). We analyzed blood samples throughout the trial at four time points. Cycle 0 (C0) reflects the baseline before the initiation of treatment, cycle 1 (C1) indicates 2 weeks after the initiation of oral daily VA and represents the effect of VA alone, cycle 2 (C2) reflects 2 weeks after the first injection of avelumab and represents the effect of VA plus one dose of avelumab, and cycles 6/7 considered as endpoint (EP) and evaluate the effects of VA plus six doses of avelumab. The response to treatment was evaluated by the iRECIST criteria [35] (based on RECIST 1.1) at the EP, which was 12/14 weeks after the trial initiation. Although our main cohort consisted of EBV and HPV‐associated carcinoma patients, we selected HPV‐associated carcinoma for this study. Based on the availability of samples for described cycles (C0, C1, C2, and EP), we collected 39 samples from 11 patients (Table 1 and Table S1). Ten additional patients, five NRs, and five Rs, from the same clinical trial, were used for the validation of plasma analytes (Table S2). The workflow of the study is illustrated in Fig. 1B and Fig. S1B. For analysis purposes, we grouped them based on their best overall clinical response assessment (iRECIST criteria) to either NR or R. The NR group consisted of iCPD (immune confirmed progressive disease), clinical progression, and iUPDs (immune unconfirmed progressive disease); however, the R group included iCCR (confirmed complete response), iCPR (confirmed partial response), and iSD (stable disease) (Table 1). The NR group was composed of 24 samples from seven patients, including C0 (*n* = 7), C1 (*n* = 5), C2 (*n* = 6), and EP (*n* = 6). The R group consisted of 15 samples from four patients, including C0 (*n* = 4), C1 (*n* = 3), C2 (*n* = 4), and EP (*n* = 4) (Table S1). To investigate the longitudinal changes and group differences, we also compared the differentially expressed transcripts in each group to their baseline (C0) and the R to the NR group at the baseline and EP, accordingly (Fig. 1C). In support of iRECIST criteria, we found a significantly higher survival probability in the R than the NR group (Fig. 1D). Of note, the survival probability was more prominent in iCCR and iCPR compared with iSD and NRs (Fig. 1E).

### 3.2 Differential transcript expression profiles of peripheral blood immune cells in Rs vs. NRs at the baseline

**Fig. 2:**
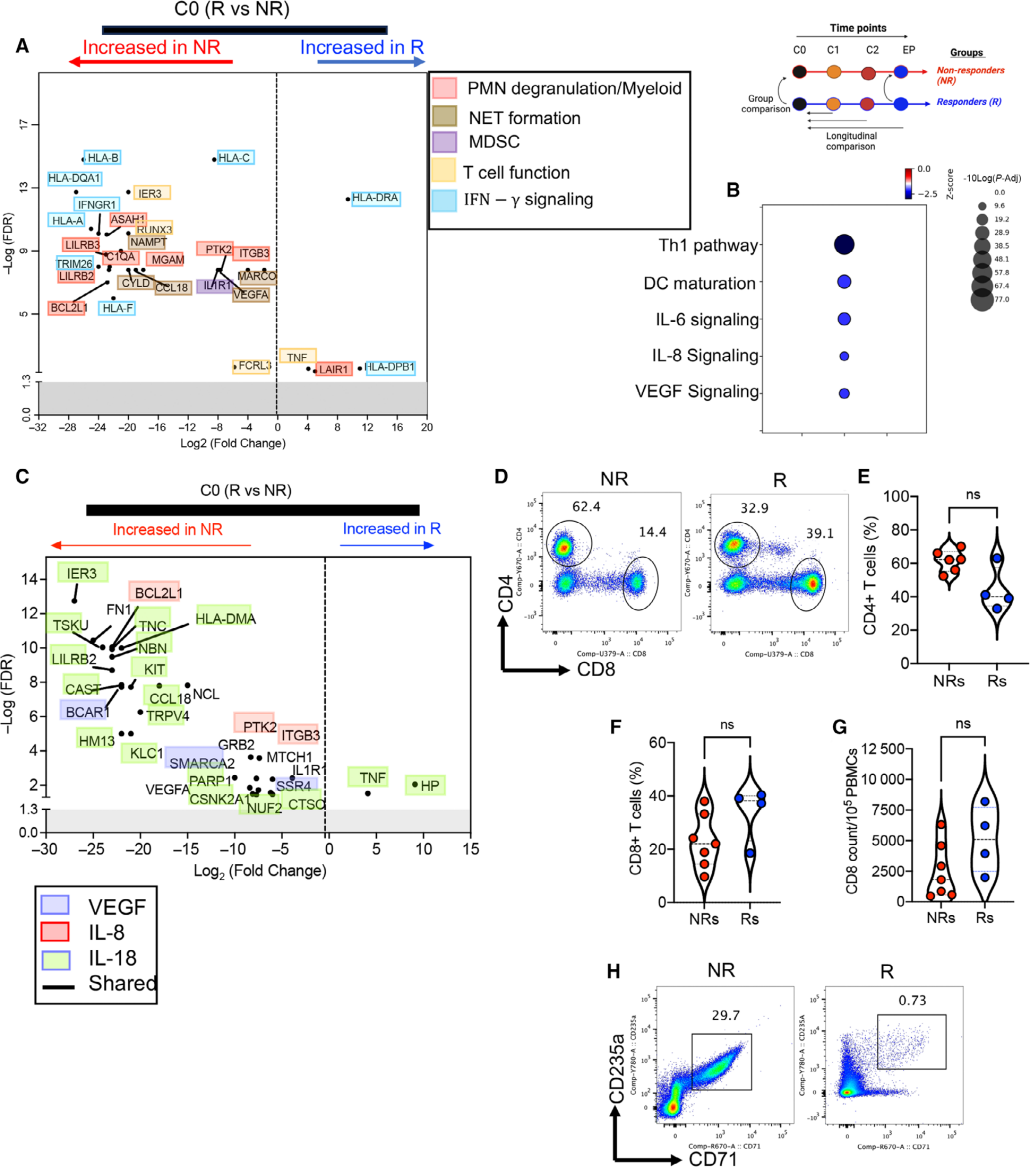
Differentially expressed transcripts, T cells, and CECs at the baseline. (A) Volcano plot showing significantly upregulated or downregulated transcripts between responders (Rs) and nonresponders (NRs) at the baseline. (B) Differentially activated pathways in Rs vs. NRs at the baseline. (C) Volcano plot showing differentially expressed transcripts in Rs compared with NRs related to VEGF, IL‐8, and IL‐18 signaling pathways at the baseline. (D) Representative flow cytometry plots and cumulative data of (E) percentages of CD4^+^ T cells, (F) percentages of CD8^+^ T cells, and (G) CD8^+^ T cells count of the peripheral mononuclear cells (PBMCs) in NRs vs. Rs at the baseline. (H) Representative flow cytometry plots of CECs in PBMCs from a NR/R. Each dot represents a patient. Not significant (ns). In Volcano plots, −log (*P*‐value) > 1.3 was considered as significant. Absolute Log_2_ fold change > 1 was considered as a threshold for up‐ or downregulation (A–C). Two‐tailed, Mann–Whitney *t*‐test ± SD (E–G). *P*‐value < 0.05 was considered as significant. The schematic above B and C indicates the comparison between NRs and Rs at cycle 0 and endpoint (EP) for panels (A–C). PMN (polymorphonuclear), NET (neutrophil extracellular trap), MDSC (myeloid‐derived suppressor cell), and DC (dendritic cell).

Based on PCA plots, we found three out of four Rs, and similarly, most of NRs, except one patient, clustered together (Fig. S1C). Then, we compared differentially expressed transcripts at the baseline. These analyses revealed 25 transcripts were upregulated and 113 transcripts were downregulated in Rs vs. NRs (Fig. S1D). When the same comparison was performed at the EP, we found 79 transcripts were upregulated and 372 transcripts were downregulated in Rs vs. NRs (Fig. S1E). Similar analyses were performed for cycles 1 and 2, which showed a differential pattern of transcripts in Rs and NRs. There were 28 upregulated and 52 downregulated transcripts at C1 but 30 upregulated and 120 downregulated transcripts at C2 in NRs vs. Rs (Fig. S1F,G). Moreover, the total number of upregulated and downregulated transcripts in Rs vs. NRs at different cycles (e.g., C0, C1, C2, and EP) are provided in Fig. S2A,B. To gain a better insight into differences at the baseline in Rs vs. NRs, we performed additional analyses. These studies revealed mostly downregulation of transcripts and pathways associated with myeloid, polymorphonuclear (PMN), and MDSCs functions in Rs vs. NRs (Fig. 2A,B). In contrast, upregulation of TNF, HLA‐II, and leukocyte‐associated immunoglobulin‐like receptor 1 (LAIR‐1) was noted in Rs (Fig. 2A). Upregulation of LAIR‐1 may inhibit myeloid cell response to different stimuli [47]. Given the enrichment of NET and neutrophil degranulation pathways in PBMCs of NRs (Fig. 2A), we suggest this could be related to the abundance of low‐density poly morphonuclear cells such as MDSCs in this group. In support of these observations, functional analysis using the IPA revealed an enriched neutrophil movement, myeloid chemotaxis in the NR at cycles 1,2, and EP (Fig. S2C) but T‐cell proliferation and activation in the R group but downregulation of neutrophils adhesion at the EP (Fig. S2D). Recently, it has been shown that biomarkers relying on signaling pathways are better predictors of response to immunotherapy [48]. Therefore, we compared the enriched signaling pathways by IPA and Metascape analysis in Rs vs. NRs at the baseline (Fig. 2C). We found transcripts related to IL‐8, IL‐18, and VEGFR (analyzed by Metascape, Reactome, and Wikipathway) pathways were downregulated in Rs but upregulated in NRs at C0 (Fig. 2C). However, TNF and haptoglobin (HP) were upregulated in Rs at the baseline (Fig. 2C). Of note, HP is reported to support T‐cell proliferation and function [49]. Thus, to determine whether there was a difference between Rs and NRs in terms of T‐cell proportions at the baseline, we quantified T‐cell frequencies in fresh PBMCs. However, we did not observe any significant difference in the proportion of CD4^+^ and CD8^+^ T cells between the groups (Fig. 2D–G and Fig. S2E). Likewise, the frequency of NK cells remained unchanged between Rs and NRs (Fig. S2F). However, the functionality of NK cells remains impaired due to the upregulation of Gal‐9 as we reported elsewhere [33]. Of note, we found that NRs have a higher frequency of CECs in their PBMCs at the baseline (Fig. 2H) as reported elsewhere [50]. In summary, NRs exhibit a phenotype enriched with PMN‐derived transcripts and genes associated with activated MDSCs and IL‐8/IL‐18 pathways at the baseline.

### 3.3 Differential transcript expression profiles of peripheral blood immune cells in NRs and Rs in different cycles

**Fig. 3:**
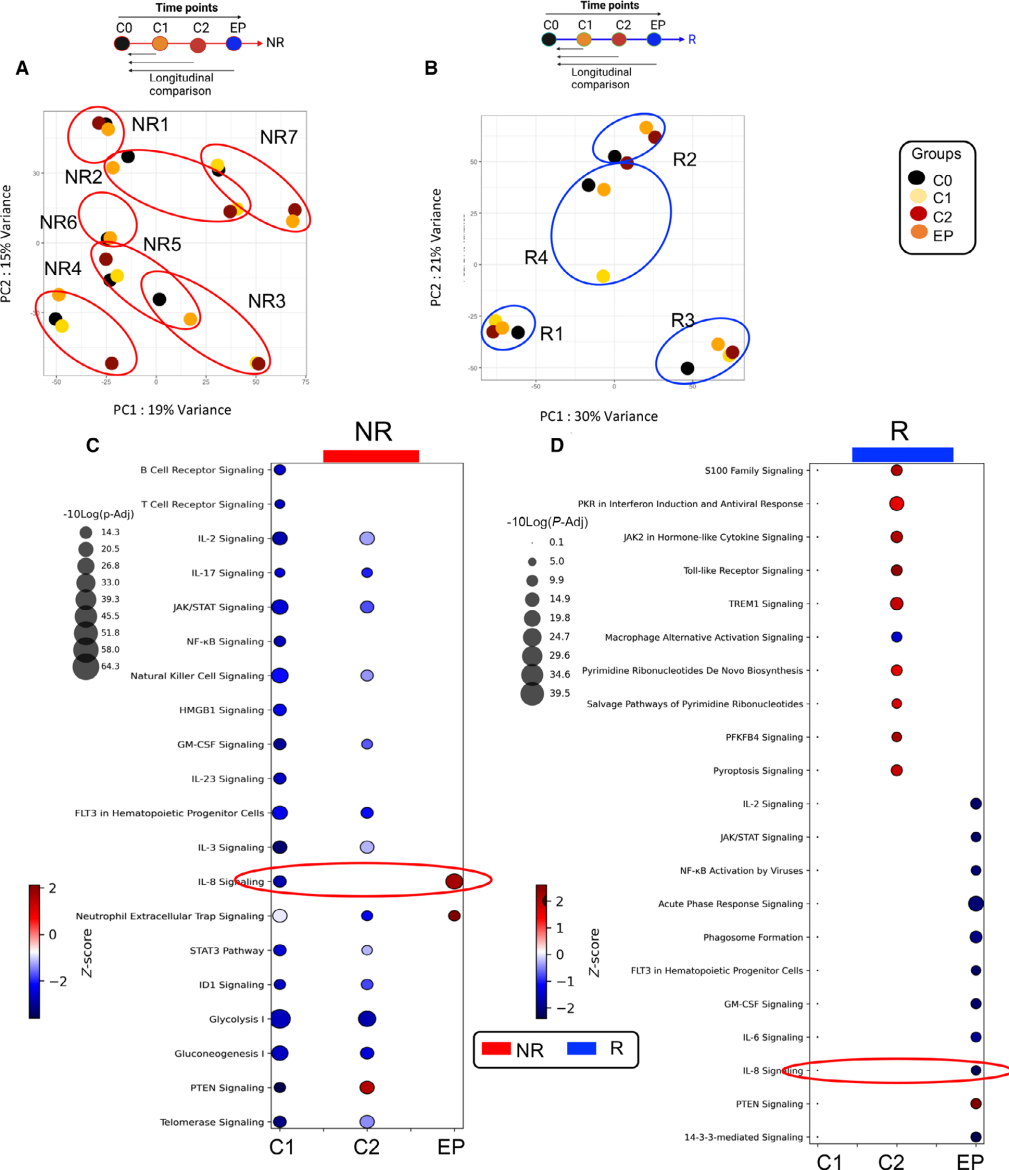
Longitudinal comparison analysis of different time points compared with the baseline. (A) Principal component analysis (PCA) plots on the Euclidian distances between nonresponders (NRs) in different time points, black dot represents cycle 0 (C0), tan dot cycle 1 (C1), dark red cycle 2 (C2), and blue endpoint (EP). (B) PCA plot on the Euclidian distances between responders (Rs) in different time points, black dot represents (C0), tan dot (C1), dark red (C2), and blue (EP). (C) Enriched signaling pathway analysis comparing C1, C2, and EP to C0 in NRs. (D) Enriched signaling pathway analysis comparing C1, C2, and EP to C0 in Rs. Threshold of Z‐score which predicts the direction of change for the function was set to an absolute value of *Z*‐score > 1.5 (blue represents downregulation and red represents upregulation). In the bubble plots for the purpose of presentation, *Z*‐score multiplied by 10. −log (*P*adj) > 1.3 considered as significant. For Volcano plots, −log (*P*adj) > 1.3 and absolute log_2_ fold change > 2 considered as significant. For transcript heatmaps, (3e‐g) log (*P*adj) < 0.05 were considered as significant and absolute log_2_ fold change > +1.5 and < −1.5 were considered as upregulated (red) or downregulated (blue). The red circle shows IL‐8 signaling in NRs and Rs.

Euclidean distances from the regularized logarithmic transformation (rlog) of the count data were calculated in all samples (Fig. S2G). To evaluate transcriptional changes following VA, avelumab, and combo treatment, each sample was compared with their C0 pairwise manner (e.g., C1 to C0, C2 to C0, and EP to C0). In brief, each group showed differential changes in their transcriptome following treatment. When C1 was compared with C0 in the NR group, we found 540 transcripts were significantly upregulated and 854 transcripts were downregulated (Fig. S3A,B). These changes appeared to be more significant at C2 compared with C0 with 941 transcripts upregulated and 790 transcripts downregulated (Fig. S3C,D). When the EP was compared with C0, we noted only 136 and 159 transcripts were up‐ and downregulated in the NR group, respectively (Fig. S3E,F). To better visualize the dispersion of whole transcripts in different cycles in the NR group, we used PCA plots. These analyses showed a minimal overlap of transcripts between patients (Fig. 3A). Moreover, the upset plots illustrate the differentially expressed transcripts and down‐ or upregulated in different cycles in the NR group (Fig. S3G,H). Interestingly, we found only a small portion of transcripts were changed after the VA treatment in the R group; 16 were upregulated vs. 11 were downregulated (Fig. S3I,J). However, once the R group was placed on avelumab, we observed substantial changes at the transcriptional level in their PBMCs compared with the baseline (Fig. S3K,L). Finally, we noted a moderate change in transcripts of the R group at the EP compared with the baseline (~ 200 transcripts were up‐ or downregulated) (Fig. S3M,N). By deploying the PCA plots, we found negligible overlap between R patients (Fig. 3B). Finally, by using the upset plots we compared transcriptional changes between C1, C2, and EP in the R group (Fig. S4A,B).

Collectively, comparing both groups to the baseline, while NRs showed a substantial change at the transcriptional levels at C1 and C2, Rs revealed slight changes at C1 but more changes at C2.

### 3.4 Valproic acid downregulated JAK/STAT and glycolysis canonical pathways in NRs

**Fig. 4:**
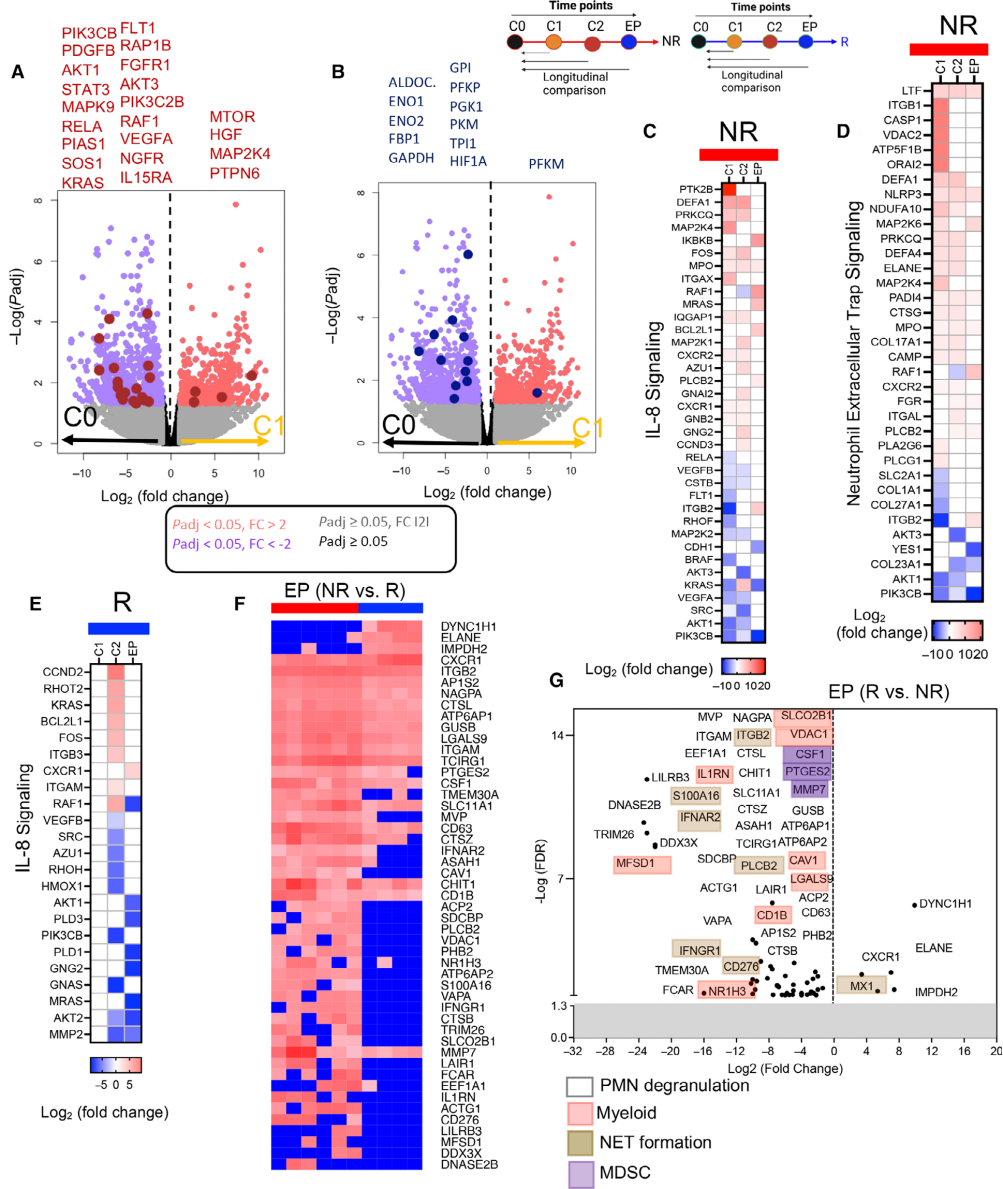
Group comparison of Rs vs. NRs at C1, C2, and endpoint. (A) Volcano plot showing differentially expressed transcripts in cycle 1 (C1) compared with cycle 0 (C0) related to JAK/STAT and STAT3 pathways, and (B) glycolysis, gluconeogenesis I pathways in nonresponder (NR) group. (C) Heatmap comparing differentially expressed transcripts in C1, C2, and endpoint (EP) compared with C0 related to IL‐8 signaling pathway, and (D) neutrophil extracellular trap (NET) formation in nonresponders (NRs). (E) Heatmap comparing differentially expressed transcripts in C1, C2, and EP compared with C0, related to IL‐8 signaling pathway in Rs. (F) Heatmap depicting differentially expressed transcripts related to genes of myeloid cells in Rs vs, NRs at the EP. (G) Volcano plots showing differentially expressed transcripts related to polymorphonuclear (PMN) degranulation, myeloid, myeloid‐derived suppressor cells (MDSC), and NET formation in Rs vs. NRs at the EP. The schematics above B and C panels indicate the comparison between C1, C2, and EP with C0 for either NRs or Rs (all panels).

As an HDAC inhibitor, VA exerts epigenetic effects by modulating nonhistone and histone proteins [25], and it targets various cell types based on their HDAC activity [51]. To investigate the biological effects of VA on immune cells, the transcriptome profile of PBMCs of NR and R groups in C1 (2 weeks post‐VA treatment) was compared with the baseline (C0). The enriched signaling analysis was generated using IPA. Differentially expressed transcripts of each cycle were analyzed for metabolic, apoptosis, cellular immune responses, humoral immune responses, cytokine signaling, and canonical transcription pathways. The results of comparative analysis comparing the differential enrichment in NRs and Rs in different cycles vs. C0 analyzed with IPA and Metascape are shown in (Fig. 3C,D, Fig. S4C,D, and Table S3A,B). We found 20 pathways were enriched as indicated in the NR group at C1 vs. the baseline (Fig. 3C, Table S3A,B). Given a small subset of differentially expressed transcripts in the R group at C1 vs. the C0 (Fig. S3I,J), we did not detect any enriched signaling pathway at C1 in this group following treatment with VA (Fig. 3D and Table S4A,B). However, a wide range of pathways was significantly downregulated in the NR group such as B‐cell receptor, T‐cell receptor, NK cell signaling, and IL‐2 signaling (Fig. 3C and Table S3A,B). Moreover, the Janus‐kinase/signal transducer and activator of transcription (JAK/STAT) signaling pathway were downregulated, and subsequently, cytokines/chemokines associated with this pathway (e.g., IL‐2, IL‐17, IL‐23, IL‐3, GM‐CSF, and FLT‐3) were downregulated [52]. Notably, IL‐8 signaling was downregulated as it binds to CXCR1/CXCR2 on the surface of neutrophils and transduces STAT3 activation through PI3K and JAK2 [53]. Most transcripts of genes involved in JAK/STAT/STAT3 pathways were downregulated in C1 compared with C0 except MAP2K4, HGF, MTOR, and PTPN6 (Fig. 4A and Table S5A). Moreover, we found metabolic pathways including glycolysis and gluconeogenesis were inhibited in the NR group at C1 (Fig. 3C and Table S3A,B) as illustrated by the downregulation of transcripts of genes related to ENO1, ENO2, ALDO, and HIF‐1α transcripts as key regulators of these pathways [54] (Fig. 4B and Table S5B). We also assessed differentially expressed transcripts related to T‐ and B cells, which confirmed the downregulation of several transcripts at C1 compared with the baseline in the NR group as shown in the volcano plot (Fig. S5A, and Table S5C,D). However, transcripts related to PLCG1, MAP2K4, MAP2K6, MAP3K8, MTOR, IGLV9‐49, IGHV3‐73, and PTPN6 genes were upregulated at C1 compared with C0 in the NR group (Fig. S5A and Table S5C,D). We also noted the downregulation and upregulation of a variety of transcripts associated with myeloid cells and NK cells at C1 compared with the baseline in this group (Fig. S5B and Table S5E,F). This is in agreement with the reported effects of VA on myeloid cells and MDSCs [55]. In summary, our observations reveal the suppressive effects of VA on different signaling pathways associated with immune cell function.

### 3.5 The activation of myeloid cells following the first dose of anti‐PD‐L1 therapy in Rs

PD‐L1 blockade has been associated with enhanced inflammatory properties of myeloid cells and a greater antigen presentation in dendritic cells (DCs) [56]. To investigate the immediate effects of avelumab on PBMCs, we analyzed the differential expression of transcripts at C2 compared with the baseline. Pathway enrichment analysis using the IPA demonstrated the significant upregulation of several pathways associated with myeloid cell activation and inflammation, including TREM1, Toll‐Like receptor (TLR), PKR in interferon induction and antiviral response, JAK‐2 in hormone‐like cytokine signaling, and S100 family signaling (Fig. 3D and Table S4A,B). Transcripts of TLR1, TLR2, TLR10, ITGB1, S100A9, S100A12, NLRP3, STAT1, and MYD88 were upregulated and IFNGR1, MMP19, MMP2, and NFKBI were downregulated at C2 compared with the baseline, which shows modifications in the innate immune response (Fig. S5C). Importantly, we observed the upregulation of metabolic signaling pathways, including the pyrimidine ribonucleotide de novo biosynthesis, salvage pathway of pyridine ribonucleotides, and PFKFB4 (6‐phosphofructokinase/fructose‐2,6‐biphosphatase 4) signaling pathway at C2 in the R group (Fig. 3D and Table S5A,B). The related transcript changes are shown in Fig. S6A. These pathways may provide metabolic intermediates as essential resources for RNA and DNA synthesis as well as plasma membrane elements for the activated myeloid cells [57]. We also noted the upregulation of the Pyroptosis pathway at C2 in the R group (Fig. 3D), which indicates programmed cell death in macrophages, DC, neutrophils, and the release of inflammatory cytokines [58].

Moreover, we noted an enriched transcriptome signature for the erythroid progenitors [59, 60] in NR but not in the R group (Figs S6B,C and S2E). These observations indicate that the expansion of CECs [5, 50, 61] may impair T‐cell effector functions in NRs as we have reported elsewhere [50, 62]. Collectively, these observations support the activation of the innate immune response, mainly myeloid cells, following the anti‐PD‐L1 therapy in the R group.

### 3.6 IL‐8 signaling pathway is activated in NRs at the endpoint

Comparing EP to the baseline, we found enrichment of pathways associated with IL‐8 signaling and neutrophil extracellular trap formation (NET) in the NR group (Fig. 3C and Table S3A,B). In contrast, IL‐8 signaling was significantly inhibited in the R group at the EP (Fig. 3D and Table S4A,B). The main transcripts associated with NET and IL‐8 signaling pathways are illustrated in NR and R groups (Fig. 4C–E and Table S5G). These results demonstrate the upregulation of various transcripts associated with IL‐8 signaling and NET formation in the NR group at C1, C2, and the EP (Fig. 4C,D, and Table S5G). For instance, transcripts of NLRP3, RAF1, MAP2K6, and ITGB2 related to NET signaling, and BCL2L1, MRAS, MPO, and IKBKB related to IL‐8 signaling were upregulated but YES1, COL23A1, PIK3CB, and KRAS were downregulated at the EP. A limited number of transcripts associated with IL‐8 signaling were significantly upregulated in the R group at C2; however, at the EP, only CXCR1 was upregulated (Fig. 4E). Overall, the transcriptional profile of NR was different from the R group at the EP (Fig. 4F) and most transcripts associated with PMN degranulation, myeloid/MDSCs, and NETosis were downregulated in the R group at the EP (Fig. 4G). These results imply that upregulation of IL‐8 signaling and myeloid cell functions are associated with poor clinical response to avelumab in NRs.

### 3.7 Immune cell population and abundance

**Fig. 5:**
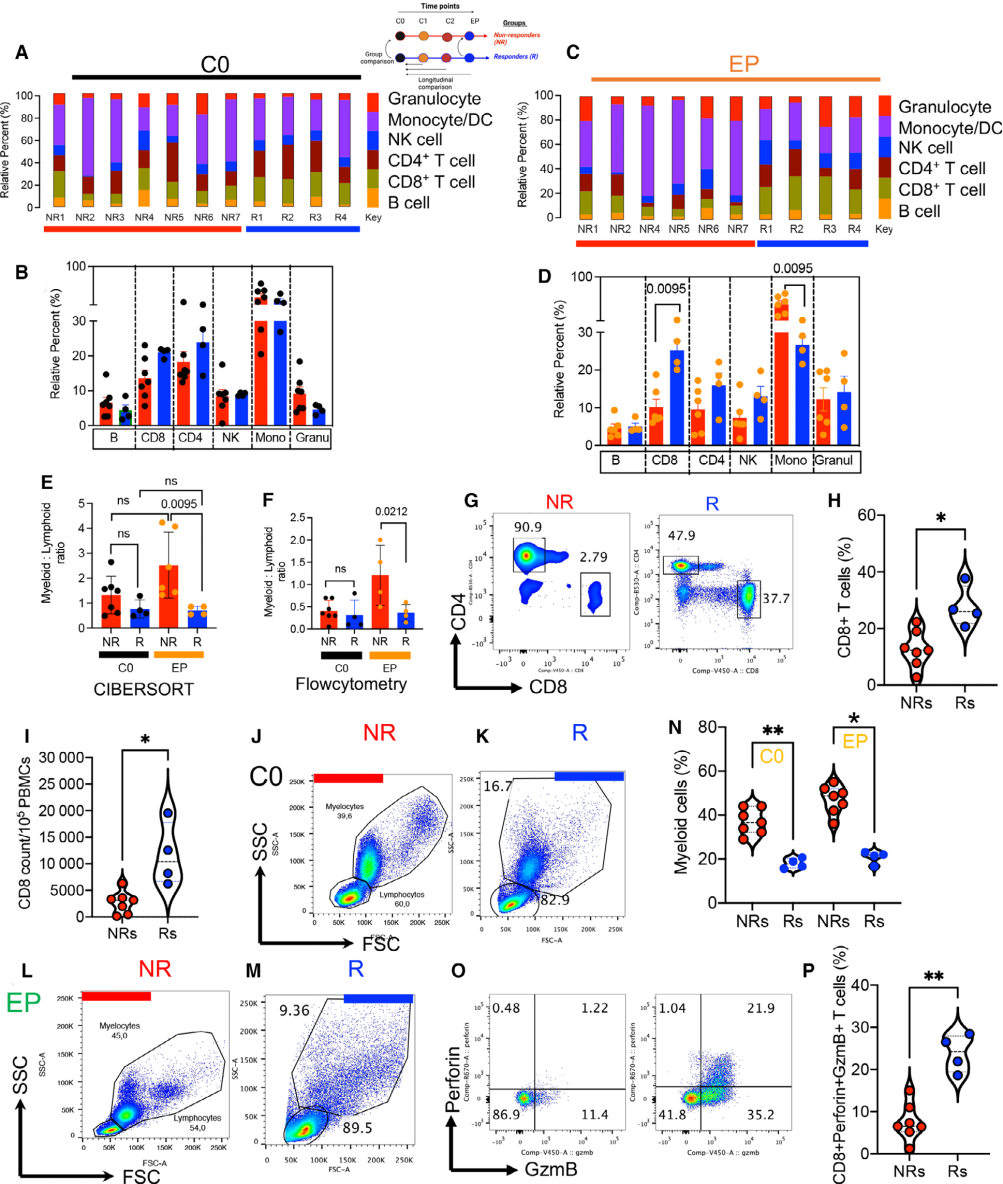
Immune cell composition in Rs and NRs at the baseline and endpoint. (A) Bar charts representing relative percentages of immune cell composition in NRs and Rs at C0. (B) Cumulative data comparing the frequency of six types of immune cells in Rs (blue) and NRs (red) at C0. (C) Bar charts representing relative percentages of immune cell compositions in NRs and Rs at the EP. (D) Bar charts comparing the frequency of six types of immune cells in Rs and NRs at the EP. (E) Cumulative data of myeloid‐to‐lymphoid ratios in Rs and NRs at the C0 and EP acquired by CIBERSORTx. The immune cell compositions acquired by importing TPM values of RNA‐Seq data to CIBERSORTx and deconvoluted to LM22 reference signature matrix. (F) Cumulative data showing myeloid‐to‐lymphoid ratios in Rs and NRs at C0 and EP obtained by flowcytometry. (G) Representative flow plots of T cells, (H) percentages of CD8^+^ T cells, and (I) CD8^+^ T‐cell count in NRs vs. Rs at the endpoint. (J) Representative flow plots showing lymphocyte and myelocyte populations in a NR, and (K) in a R at C0. (L) Representative flow plots showing lymphocyte and myelocyte populations in a NR, (M) in a R at the EP. (N) Cumulative data of frequency of myeloid cells at the Co and EP in NRs and Rs. (O) Representative flow cytometry plots and (P) cumulative data of GzmB/perforin expression in CD8^+^ T cells from NRs vs. Rs at the EP. *P*‐value was determined using ANOVA (B, D–F) or two‐tailed, Mann–Whitney *t*‐test ± SD (H, I, N, P). *P*‐value < 0.05 was considered as significant. *P*‐value < 0.05 was considered as significant. *P < 0.05, **P < 0.01.

To determine the relative frequency of different immune cells in the bulk RNA‐Seq, we carried out the CIBERSORTx analysis to deconvolute the estimated 22 types of immune cells (LM22) [43] (Table S6). To simplify our data analysis, 22 cell subsets were merged into six main immune cell lineages: B cells, CD8^+^ T cells, CD4^+^ T cells, NK cells, monocytes, and granulocytes. Comparing the relative percentages of immune cell subsets between groups at the baseline, we did not notice any significant difference between Rs and NRs despite a higher trend of enriched CD8^+^ and CD4^+^ T cells in Rs (Fig. 5A,B). Similarly, there was no significant difference between the groups at C1 and C2 (Fig. S7A,B). Although we observed significantly higher percentages of CD8^+^ T cells in PBMCs of Rs, the monocyte fraction was significantly enriched in the NR group (Fig. 5C,D). In agreement, we found that the myeloid (monocytes and granulocytes) to lymphoid (B, CD8^+^ T, CD4^+^ T, and NK cells) ratio was significantly higher in NRs at the EP (Fig. 5E). The variation in immune cell subsets was noted over times as analyzed by the CIBERSORTx (Fig. S7C,D). Moreover, we validated our analyzed data by the CIBERSORTx using flow cytometry (Fig. 5F–N). These observations confirmed the expansion of myeloid cells in NRs (Fig. 5N) but higher frequency of CD8^+^ T cells in Rs at the EP. We observed not only Rs have a higher proportion of CD8^+^ T cells but also these cells exhibit a greater perforin/granzyme B (GzmB) expression at the EP (Fig. 5O,P). Collectively, the estimation of immune cell fractions distinguishes Rs from NRs at the endpoint, as the R group had a lower myeloid‐to‐lymphoid ratio. The myeloid‐to‐lymphoid ratio agrees with reports that a higher myeloid cell/MDSCs to lymphoid ratio is accompanied by a worse prognosis in cancer [63].

### 3.8 Plasma IL‐8/IL‐18 concentrations and IL‐8/IL‐18 signaling pathways discriminate NRs from Rs at the baseline

**Fig. 6:**
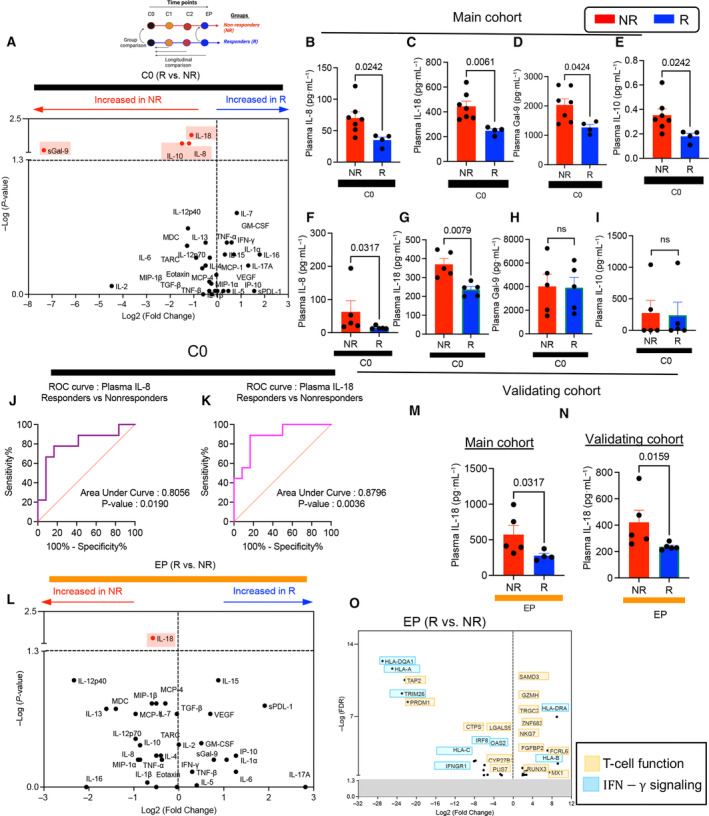
Soluble mediator analysis in Rs and NR at the baseline and endpoint. (A) Volcano plot illustrating the significance and magnitude of differences in soluble mediators in responders (Rs) vs. nonresponders (NRs) at cycle 0 (C0). (B) Cumulative data of the plasma concentration of IL‐8, (C) IL‐18, (D) Gal‐9, and (E) IL‐10 in NRs and Rs of the main cohort at C0. (F) Cumulative data of the plasma concentration of IL‐8, (G) IL‐18, (H) Gal‐9, and (I) IL‐10 obtained from 10 patients of the validation cohort at C0. (J) The receiver operator characteristic (ROC) curve of comparison between plasma IL‐8 in Rs and NRs. (K) The ROC curve of comparison between plasma IL‐18 in Rs and NRs. (L) Volcano plot illustrating the significance and magnitude of the differences in soluble mediators in Rs vs. NRs at the endpoint (EP). (M) Cumulative data of the plasma concentration of IL‐18 in the main cohort and (N) the validating cohort at the EP. (O) Volcano plot showing differentially expressed transcripts related to T‐cell function and IFN‐γ signaling in Rs compared with NRs. Volcano plots ‐log (*P*‐value) > 1.3 was considered as significant. Absolute Log_2_ fold change > 1 was considered as threshold for up‐ or downregulation. *P*‐value < 0.05 was considered as significant. Two‐tailed, Mann–Whitney *t*‐test ± SD (B–I, M, N) or receiver operating characteristic curve analysis (J, K). *P*‐value < 0.05 was considered as significant. Not significant (ns).

Cytokines and chemokines secreted by tumor, stromal, and immune cells play crucial roles in shaping and orchestrating immune responses against tumors [64]. To investigate the cytokine and chemokine profiles in Rs vs. NRs, we measured 28 analytes including IFN‐γ, IL‐1β, IL‐2, IL‐4, IL‐6, IL‐10, IL‐12p70, IL‐13, TNF‐α, GM‐CSF, IL‐1α, IL‐5, IL‐7, IL‐12p40, IL‐15, IL‐16, IL‐17A, TNF‐b, VEGF, eotaxin, MIP‐1α, TARC, IP‐10, MIP‐1β, IL‐8, MCP‐1, MDC, and MCP‐4 in the plasma of patients at C0, C1, and EP. In addition, the plasma was subjected to Gal‐9, IL‐18, soluble PD‐L1, and TGF‐β ELISAs. At the baseline, IL‐8, IL‐18, Gal‐9, and IL‐10 were significantly elevated in NRs compared with Rs (Fig. 6A–E). However, the concentration of other measured analytes remained comparable (Fig. 6A). The concentration of these four analytes was measured in a validating cohort consisted of 10 patients including five NRs and five Rs (Table S2). In agreement, IL‐8 and IL‐18 concentrations were significantly higher in NRs (Fig. 6F,G), but this was not the case for Gal‐9 and IL‐10 (Fig. 6H,I). Then, we hypothesized that plasma IL‐8/IL‐18 levels could be used as biomarkers to stratify potential NRs from Rs as a predictive strategy. Therefore, we used the receiver operator characteristic (ROC) curve to estimate the best cutoff values of the plasma IL‐8/IL‐18 at the baseline, as we have reported elsewhere [65]. The area under the curve to discriminate NRs from Rs for IL‐8 and IL‐18 was 0.8056 and 0.8796, respectively (Fig. 6J,K). The cutoff value of 20.77 pg·mL^−1^ for IL‐8 and 311.2 pg·mL^−1^ for IL‐18 showed a test sensitivity of (75% and 88.89%) and test specificity of (76.92% and 76.92%) at C0, respectively. However, following VA treatment, the concentrations of soluble markers remained unchanged between Rs and NRs (Fig. S7E). At the EP, we found that only IL‐18 was elevated in NRs vs. Rs (Fig. 6L,M), which was reproduced in the validating cohort (Fig. 6N). Finally, we compared the concentration of soluble analytes at the EP vs. the C0 in each group. These observations showed a significant increase in the plasma concentration of IL‐1β, IL‐10, and VEGF in NRs but not in the R group over time (Fig. S8A–C). These markers are best known to be associated with poor prognostic in cancer patients [66, 67, 68]. Moreover, these cytokines are related to MDSCs development and expansion [69]. Therefore, our data indicate the elevation of plasma IL‐8 and IL‐18 at the baseline in NRs. Hence, these biomarkers could be used to discriminate NRs from Rs. Likewise, a positive link between these cytokines with poor prognostic in various cancer types has been documented [70, 71]. Recently, it has been shown that biomarkers relying on signaling pathways are better predictors of response to immunotherapy [48]. Therefore, we compared the enriched signaling pathways by IPA and Metascape analysis in Rs vs. NRs at the baseline (Fig. S8D,E). We found transcripts related to enriched IL‐8 (analyzed by IPA) and VEFG/VEGFR (analyzed by Metascape and Reactome) signaling pathway were observed in NRs at the endpoint (Fig. S9).

Our further analyses revealed downregulation of IL‐8, NET formation, and other myeloid‐related signaling pathways in Rs but in contrast upregulation of signaling pathways associated with adaptive immunity and leukocyte activation in Rs at the endpoint (Fig. S8D,E).

Finally, we found mostly upregulation of transcripts associated with T‐cell function but downregulation of transcripts associated with IFN‐γ signaling in Rs. Of note, the restriction of FC receptor‐like (FCRL6) expression in CD8^+^ T cells has received significant attention in immune checkpoint blockers [72], and RUNX2/3 transcriptional factors are required for tissue‐resident memory T‐cell localized immunity [73]. Also, we noted the upregulation of natural killer cell granule protein 7 (NKG7) in Rs at the endpoint, which is involved in enhanced cytolytic activity of CD8^+^ T cells [74]. In contrast, we observed the downregulation of Gal‐9 in Rs at the endpoint (Fig. 6O).

## 4 Discussion

This study demonstrates the prognostic value of circulating immune transcriptome and plasma cytokine levels in HPV‐associated squamous cell carcinoma patients at the baseline and throughout the treatment. NRs had elevated basal plasma IL‐8/IL‐18 levels and activated IL‐8/IL‐18 signaling pathways in their peripheral blood immune cells. Following treatment at the endpoint, the NR group showed further upregulation of IL‐8 signaling accompanied by the activation of the NET formation pathway and elevated levels of plasma IL‐18. The IL‐8 signaling pathway is linked to the chemoattraction of MDSCs, neutrophils, and other myeloid cell populations to the TME. Similarly, IL‐18 is associated with M‐MDSC induction [75, 76]. As such, our observations imply that the enhanced IL‐8/IL‐18 signaling by the accumulation of MDSCs may contribute to an immune nonresponsiveness to ICIs (e.g., avelumab).

In line with this hypothesis, deconvoluted data with digital cytometry (CIBERSORTx) [43] and flow cytometry analysis revealed a higher myeloid‐to‐lymphoid ratio in NRs compared with Rs at the endpoint. Extracted myeloid transcriptome in NR and Rs revealed prohibitive myeloid expression in NRs with predominant neutrophil‐related transcripts such as granule and NET formation. For example, CSF1, IL1R1, MMP7, and PTGES2 related to myeloid suppressive functions were upregulated in NRs, as reported elsewhere [77]. Myeloid compartment enrichment following avelumab therapy might be a resistance strategy mechanism in NRs. In agreement with our results, previous studies have shown the capacity of myeloid cells/MDSCs to arbitrate resistance and poor prognosis in gastric cancer [78]. Although human neutrophils and PMN‐MDSCs possess similar phenotypes, neutrophils are located in the high‐density gradient portion, whereas PMN‐MDSCs are present with PBMCs as light density when subjected to cell isolation [12]. Given our methodology of subjecting PBMCs to RNA‐Seq, the saturated neutrophil signature in NRs reflects PMN‐MDSCs. However, further studies are required to determine the effector functions of these cells [12].

This myeloid signature was associated with an enhanced NET formation pathway and elevated IL‐8/IL‐18 in NRs at the endpoint. IL‐8 (CXCL8) is a chemokine released by tumor, stromal, and myeloid cells to attract myeloid cells to the tumor tissue [79] and is involved in angiogenesis, tumor progression, and reduced response to ICIs [80, 81]. Similarly, IL‐18 is linked to poor prognosis as an immunosuppressor cytokine in cancer [75] and is reported to be elevated in NRs to ICIs [82]. Our data support the sensitivity and specificity of plasma IL‐8 and IL‐18 as predictive biomarkers in NRs. A similar correlation between plasma IL‐8 and poor immune response to anti‐PD‐L1 therapy has been reported [81]. However, the implication of plasma IL‐18 as a prognostic biomarker in cancer has been a subject of debate [70] and merits further investigations.

Moreover, other cytokines such as IL‐6, IL‐1β, VEGF, and IL‐10 have been correlated with poor prognosis in cancer patients due to their role in regulating myeloid cell (e.g., MDSCs) differentiation and recruitment to the tumor [83, 84]. IL‐6 expression is upregulated by E6 and E7 proteins from high‐risk HPVs that have been related to carcinogenesis [85]. Likewise, we observed an increase in plasma IL‐1β, IL‐10, and VEGF in our NRs cohort, which was associated with an enhanced VEGF/VEGFR signaling pathway [86]. Of note, IL‐10 in high‐risk HPV infection is associated with tumor formation and progression [87, 88]. Although IL‐10 and Gal‐9 were elevated in the plasma of NRs at the baseline, our validating cohort did not verify these observations. Therefore, further studies in larger cohorts are needed to clarify the role of soluble Gal‐9 in virus‐associated cancers considering its role in T‐cell exhaustion [33]. It is worth mentioning that we did observe the downregulation of Gal‐9 transcripts in Rs at the endpoint. Given the role of Gal‐9 in T‐cell exhaustion and NK cell dysfunction [89, 90], it is likely to speculate that increased Gal‐9 expression in NRs may negatively impact T‐/NK cell effector functions. The upregulation of NKG7 in Rs may imply enhanced degranulation and cytotoxic ability of CD8^+^ T cells [74], and upregulation of RUNX could promote the migratory capacity and cytotoxicity of tissue‐resident memory CD8^+^ T cells [73].

Recent evidence unveils a role for NET formation in cancer progression and metastasis in animal cancer models [91]. NETs cover cancer cells and protect them from the immune system [92]. In this scenario, IL‐8 is important in attracting myeloid cells/MDSCs, resulting in enhanced NET formation in the tumor [76] and decreasing the clinical benefit of ICIs [80].

In this study, we treated patients with VA 2 weeks before initiating avelumab. We aimed to determine the immunomodulatory effects of this HDAC inhibitor [93]. HDAC inhibitors target different classes of HDAC enzymes, and their effects are selective and depend on the enzymatic activity of HDACs in different cells. For example, entinostat selectively targets PMN‐MDSCs and modulates their functions [94]. Due to the selective property of HDAC inhibitors, the immunomodulatory role of VA in cancer requires further investigation. In our cohort, VA combined with anti‐PD‐L1 disclosed a substantial difference in signaling dynamics between NRs and Rs. The downregulated signaling pathways such as HIF‐1α and glycolysis, JAK/STAT, and STAT3 favor immune system deactivation. HIF‐1α induces genes related to the glycolytic pathway, and enhanced glycolysis supports MDSCs' expansion and function [95]. Similarly, STAT3 signaling plays a complex role in immune cell growth, differentiation, and apoptosis, and is involved in the negative regulation of immune response, mainly regulated by IL‐6 through JAK/STAT signaling [52]. In general, VA modulates both innate and adaptive immune cells [32]. It has been shown to induce T‐cell apoptosis [96], modulate NK cells, DCs, and macrophages, and regulate the inhibitory functions of MDSCs [97]. In particular, VA inhibits HDAC class I (HDAC 1, 2, 3) and impairs the immunosuppressive function of PMN‐MDSC [55]. In support of this possibility, it has been reported that VA enhances anti‐PD‐L1 immunotherapy by attenuating MDSCs [98]. HDAC activity has been reported to be selective and higher in MDSCs [97]. However, the enriched MDSC signature of NRs may reduce the effectiveness of VA in NRs in our cohort. Alternatively, a higher VA dosage might be necessary to attenuate MDSCs and myeloid cell functions in NRs than Rs [55].

In contrast, we observed an activated innate immune response in the R group based on the upregulation of TREM‐1 and TLR signaling pathways. These canonical pathways are mainly related to myeloid cell functions [99] and stress response [100]. It has been shown that anti‐PD‐L1 therapy initiates a perceptible inflammatory signature in CD14^+^ monocytes with elevated levels of myeloid‐derived cytokine [56]. Moreover, an activated inflammatory myeloid cell signature with the upregulation of Heparin‐binding EGF‐like factor (HBEGF), THBS (thrombospondin‐1), IL‐1β, CXCL1, CXCL2, NLRP3, and increase in inflammasome‐associated cytokines (IL‐1β and IL‐18) have been documented upon anti‐PD‐L1 therapy [56]. Also, MDSCs upregulate PD‐L1 under hypoxia through HIF‐1α by binding to HRE (hypoxia‐response element) in the proximal PD‐L1 promotor, which provides a negative signal by attenuating their suppressive function [101]. PD‐L1 is mainly expressed in M‐MDSCs and, to a lesser extent, in PMN‐MDSCs [102]. In NRs, we did not observe substantial changes in signaling pathways at C2. The enriched presence of immunosuppressive myeloid cells may be a reason for this. On the contrary, a more inflammatory response was associated with increased frequency of functional CD8^+^ T cells in the R group. This was supported by a higher proportion of CD8^+^ T cells exhibiting a greater cytotoxicity capability (e.g., GzmB/perforin expression) in Rs at the endpoint.

We are aware of multiple study limitations. The small cohort size is our major limitation. Also, due to the poor quality of RNA, we had to exclude some samples/time points. Because of the small sample size (*n* = 4, R), we were unable to conduct statistical analysis and compare iCCR, iCPR, and stable Rs from progressive cases. There is a possibility that each subset of Rs (e.g., iCCR, iCPR, or iSD) may present a unique RNA‐Seq, plasma proteome, and immune cell phenotype. These examples encourage similar studies in larger cohort size to compare RNA‐Seq profile and immune responses in various subpopulations. Also, longitudinal follow‐up beyond the endpoint is required to differentiate long‐lasting Rs from those that may acquire resistance to immunotherapy. Moreover, we were limited with the blood volume to perform additional functional assays to better characterize cellular immune components in Rs vs. NRs.

## 5 Conclusions

Taken together, we found elevated baseline plasma IL‐8/IL‐18 levels and activated IL‐8/IL‐18 signaling pathways in NRs at the baseline. Even at the endpoint, the NR group exhibited a similar pattern of enhanced IL‐8/IL‐18 signaling accompanied by the NET formation pathway. The chemotactic effects of the IL‐8/IL‐18 signaling pathway on MDSCs, neutrophils, and other myeloid cells [75, 76] may contribute to the acquired resistance against ICIs. Moreover, we observed that VA transiently modulates hematopoiesis as evidenced by the expansion of CECs in NRs. Given the immunosuppressive nature of CECs [5, 103], their expansion may provide another immune evasion mechanism in NRs. However, it is unclear why VA exhibits differential effects in Rs vs. NRs in our cohort. This might be related to differential HDAC activity in these two subpopulations or other unknown factors.

Our findings highlight the importance of peripheral blood immune signature as a noninvasive biomarker source to characterize Rs and NRs in a clinical setting. For example, a qPCR quantification approach for IL‐8/neutrophil extracellular trap signature could serve as a prognostic biomarker in Rs vs. NRs. Such studies could be applied to larger cohorts to identify a specific immune signature for precision medicine.

## Conflict of interest

The authors declare no conflict of interest.

## Author contributions

NB performed most of the study, analyzed clinical data, performed RNA‐Seq, analyzed related data, and wrote the first draft of the manuscript. HS assisted in RNA‐Seq data analysis. SM processed blood samples for RNA‐Seq. JW designed, led the clinical trial, assessed the clinical outcomes, and provided insight. SE conceptualized the study, secured resources, supervised the study, and wrote the manuscript.

### Peer review

The peer review history for this article is available at https://www.webofscience.com/api/gateway/wos/peer‐review/10.1002/1878‐0261.13519.

## Supporting information


**Fig. S1.** Schematic RNA‐Seq analysis and heatmaps of differentially expressed transcripts at different cycles in responders (R) vs nonresponders (NRs).
**Fig. S2.** Comparison of upregulated and downregulated transcripts in Rs vs NRs at different cycles and flow cytometry gating strategy.
**Fig. S3.** Heatmaps and volcano plots of down and upregulated transcripts in different cycles in NRs and Rs.
**Fig. S4.** Transcriptional changes between C1, C2, and EP in the R group and altered pathways in NRs and Rs at cycles 1, 2, and endpoint (EP).
**Fig. S5.** CIBERSORTx analysis of RNA‐Seq transcripts related to different immune cells in NRs and Rs.
**Fig. S6.** Upregulated transcripts associated with erythroid cells in NR.
**Fig. S7.** CIBERSORTx analysis of RNA‐Seq transcripts shows the proportion of different immune cells in NR vs R at different cycles.
**Fig. S8.** Plasma cytokines/chemokines levels in NRs and Rs.
**Fig. S9.** Transcripts related to enriched IL‐8 and VEFG/VEGFR as analyzed by Metascape, Reactome in NR vs R at the endpoint.

**Table S1.** Characteristics of samples used for RNA‐Seq.

**Table S2.** HPV‐associated metastatic patients' characteristics.

**Table S3.** (A, B) Enriched pathways in the NR group at C1 versus the baseline (C0).
**Table S4.** Enriched signaling pathway at C1 vs C0 in Rs.
**Table S5.** (A) Transcripts of genes involved in JAK/STAT/STAT3 pathways in C1 vs C0 in NRs. (B) Transcripts of genes involved in glycolysis and gluconeogenesis pathways in C1 vs C0 in NRs. (C) Transcripts related to B cells at C1 compared with the baseline in the NR. (D) Transcripts related to T cells at C1 compared with the baseline in the NR. (E) Transcripts related to NK cells at C1 compared with the baseline in the NR. (F) Transcripts related to myeloid cells at C1 compared with the baseline in the NR. (G) Transcripts related IL‐8 signaling and NET formation at C1 compared with the baseline in the NR.
**Table S6.** CIBERSORTx analysis to deconvolute the estimated immune cell transcripts.

## Data Availability

All the generated data related to this study are incorporated in the main and supplemental information. In addition, original data related to RNA‐Seq are available from the SRA portal on NCBI under accession number GSE229014.
