# Supplementary material for: Transcriptomic profiling of peripheral blood cells in HPV‐associated carcinoma patients receiving combined valproic acid and avelumab
Source: Mol Oncol. 2023 Sep 17;18(5):1209–30. doi: 10.1002/1878-0261.13519 (PMC11077001; doi:10.1002/1878-0261.13519)
Supplement: Supplementary file 1 — Fig. S1. Schematic RNA‐Seq analysis and heatmaps of differentially expressed transcripts at different cycles in responders (R) vs nonresponders (NRs). Fig. S2. Comparison of upregulated and downregulated transcripts in Rs vs NRs at different cycles and flow cytometry gating strategy. Fig. S3. Heatmaps and volcano plots of down and upregulated transcripts in different cycles in NRs and Rs. Fig. S4. Transcriptional changes between C1, C2, and EP in the R group and altered pathways in NRs and Rs at cycles 1, 2, and endpoint (EP). Fig. S5. CIBERSORTx analysis of RNA‐Seq transcripts related to different immune cells in NRs and Rs. Fig. S6. Upregulated transcripts associated with erythroid cells in NR. Fig. S7. CIBERSORTx analysis of RNA‐Seq transcripts shows the proportion of different immune cells in NR vs R at different cycles. Fig. S8. Plasma cytokines/chemokines levels in NRs and Rs. Fig. S9. Transcripts related to enriched IL‐8 and VEFG/VEGFR as analyzed by Metascape, Reactome in NR vs R at the endpoint. [file MOL2-18-1209-s003.pdf]

S Fig. 1

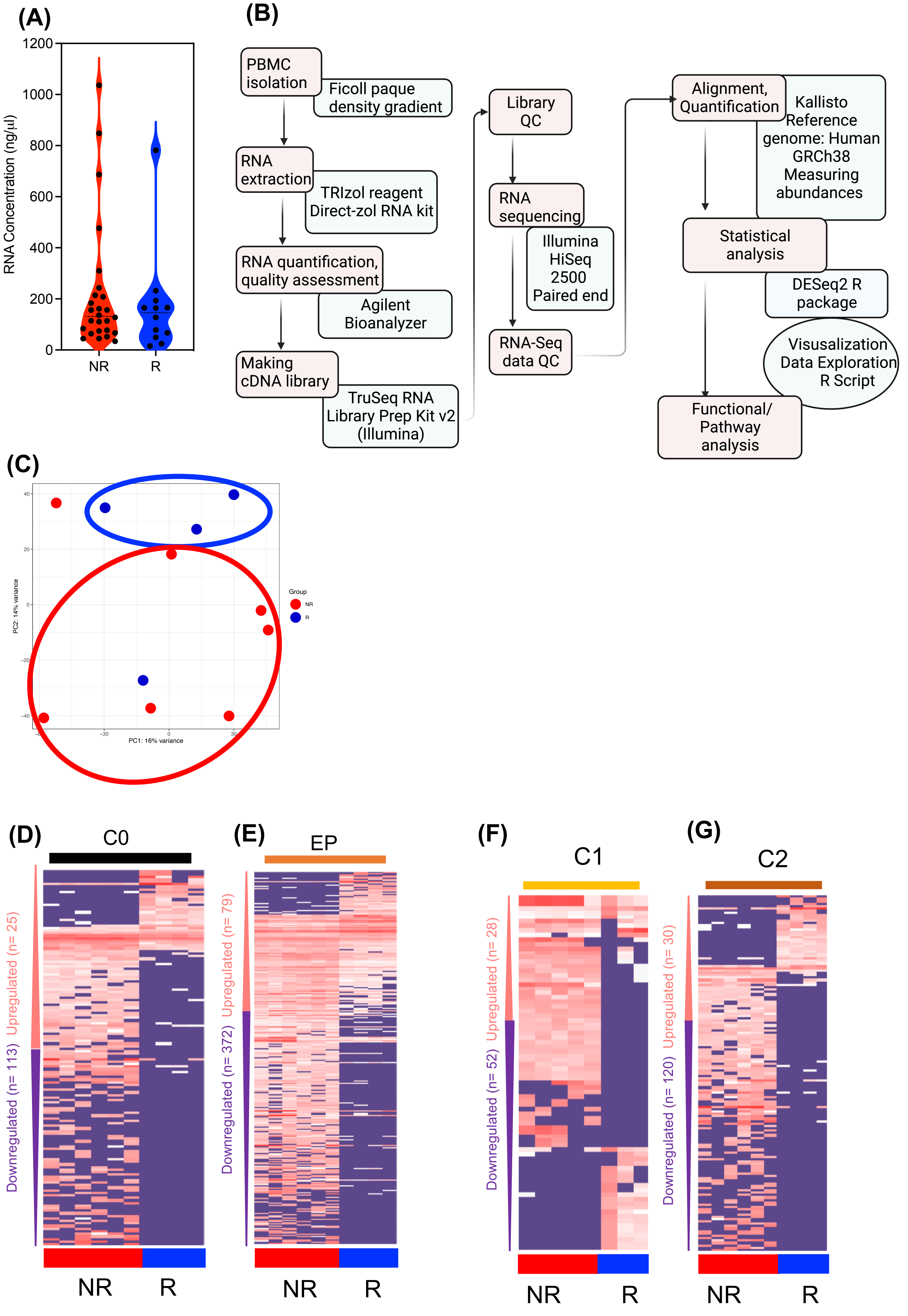
(A)
RNA Concentration (ng/µl)
1200
1000
800
600
400
200
0
NR
R
(B)
PBMC isolation
Ficoll paque density gradient
RNA extraction
TRIzol reagent Direct-zol RNA kit
RNA quantification, quality assessment
Agilent Bioanalyzer
Making cDNA library
TruSeq RNA Library Prep Kit v2 (Illumina)
Library QC
RNA sequencing
Illumina HiSeq 2500 Paired end
RNA-Seq data QC
Alignment, Quantification
Kallisto Reference genome: Human GRCh38 Measuring abundances
Statistical analysis
DESeq2 R package
Visusalization Data Exploration R Script
Functional/ Pathway analysis
(C)
PC2: 14% variance
PC1: 16% variance
Group
NR
R
(D)
C0
Upregulated (n= 25)
Downregulated (n= 113)
NR
R
(E)
EP
Upregulated (n= 79)
Downregulated (n= 372)
NR
R
(F)
C1
Upregulated (n= 28)
Downregulated (n= 52)
NR
R
(G)
C2
Upregulated (n= 30)
Downregulated (n= 120)
NR
R

S Fig. 2

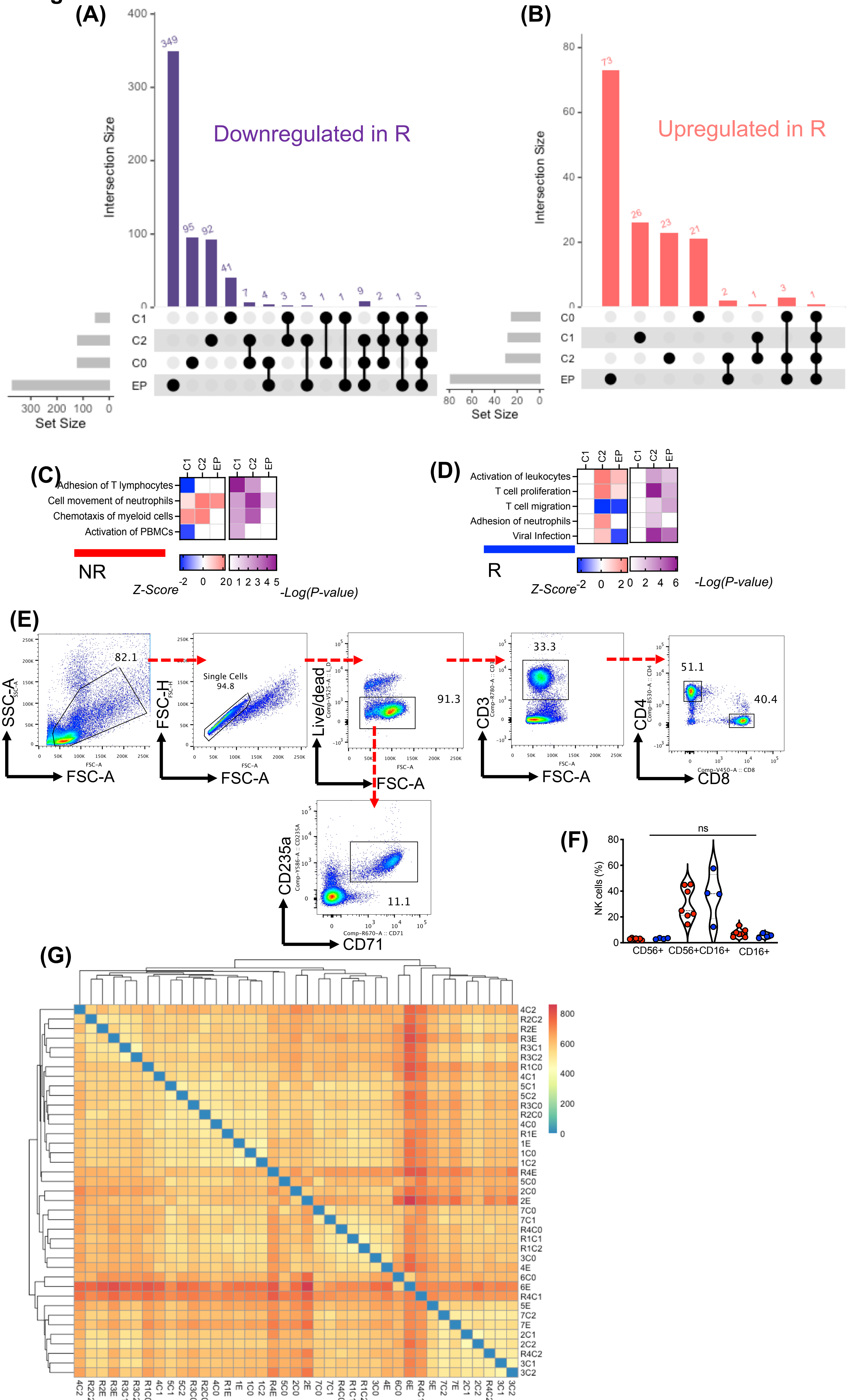

(A)
Downregulated in R
Intersection Size
Set Size
C1
C2
C0
EP
(B)
Upregulated in R
Intersection Size
Set Size
(C)
Adhesion of T lymphocytes
Cell movement of neutrophils
Chemotaxis of myeloid cells
Activation of PBMCs
NR
Z-Score
-Log(P-value)
(D)
Activation of leukocytes
T cell proliferation
T cell migration
Adhesion of neutrophils
Viral Infection
R
Z-Score
-Log(P-value)
(E)
SSC-A
FSC-A
FSC-H
Single Cells
94.8
82.1
Live/dead
91.3
CD3
33.3
CD4
CD8
51.1
40.4
CD235a
CD71
11.1
(F)
ns
NK cells (%)
CD56+
CD56+CD16+
CD16+
(G)

S Fig. 3

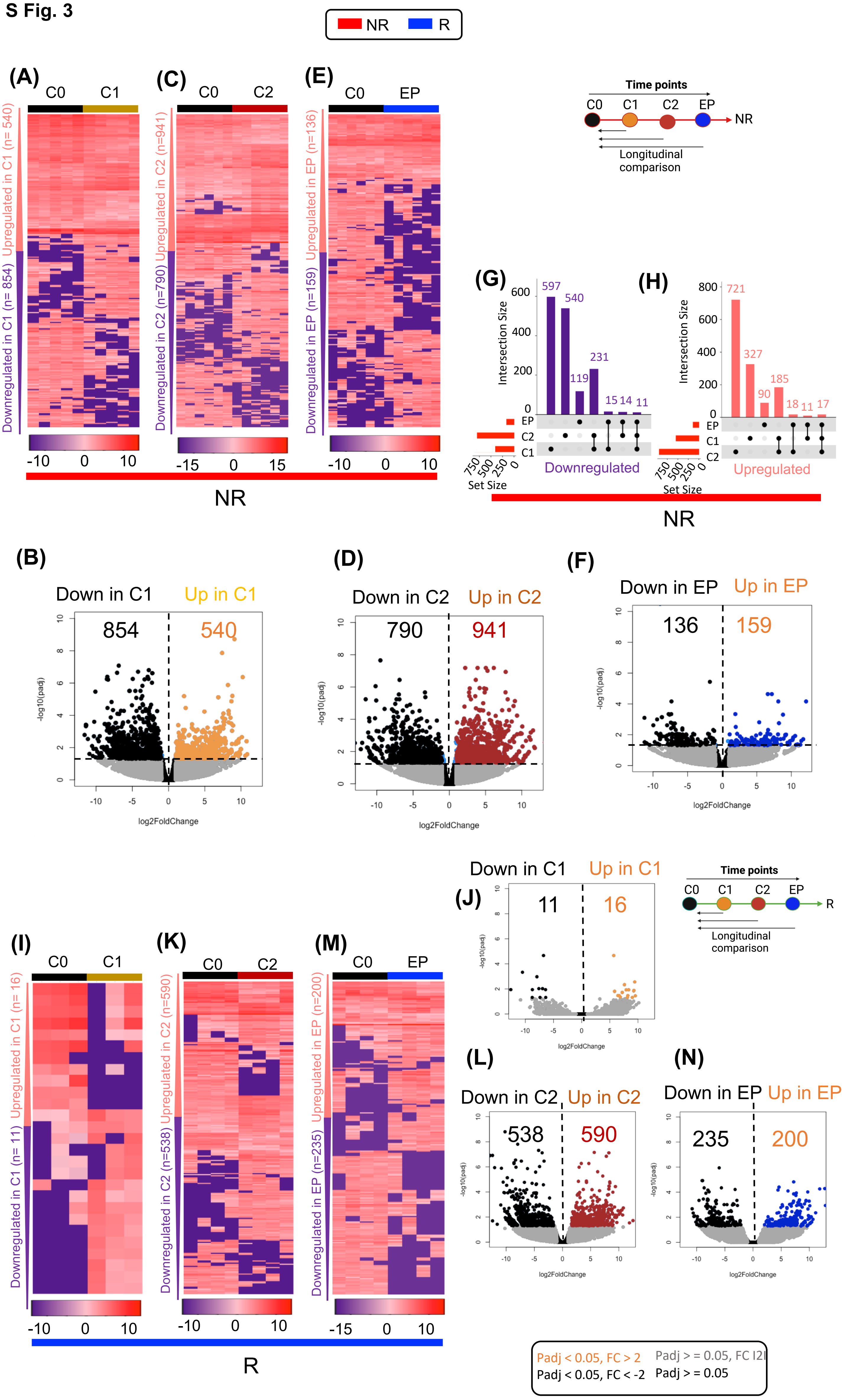
NR
R
(A)
C0
C1
Upregulated in C1 (n= 540)
Downregulated in C1 (n= 854)
-10
0
10
(C)
C0
C2
Upregulated in C2 (n=941)
Downregulated in C2 (n=790)
-15
0
15
(E)
C0
EP
Upregulated in EP (n=136)
Downregulated in EP (n=159)
-10
0
10
NR
Time points
C0
C1
C2
EP
NR
Longitudinal comparison
(G)
Intersection Size
597
540
119
231
15
14
11
EP
C2
C1
Set Size
Downregulated
(H)
721
327
90
185
18
11
17
EP
C1
C2
Set Size
Upregulated
NR
(B)
Down in C1
Up in C1
854
540
-log10(padj)
log2FoldChange
(D)
Down in C2
Up in C2
790
941
(F)
Down in EP
Up in EP
136
159
Down in C1
Up in C1
(J)
11
16
Time points
C0
C1
C2
EP
R
Longitudinal comparison
(I)
C0
C1
Upregulated in C1 (n= 16)
Downregulated in C1 (n= 11)
-10
0
10
(K)
C0
C2
Upregulated in C2 (n=590)
Downregulated in C2 (n=538)
-10
0
10
(M)
C0
EP
Upregulated in EP (n=200)
Downregulated in EP (n=235)
-15
0
10
R
(L)
Down in C2
Up in C2
538
590
(N)
Down in EP
Up in EP
235
200
Padj < 0.05, FC > 2
Padj > = 0.05, FC I2I
Padj < 0.05, FC < -2
Padj > = 0.05

S Fig. 4

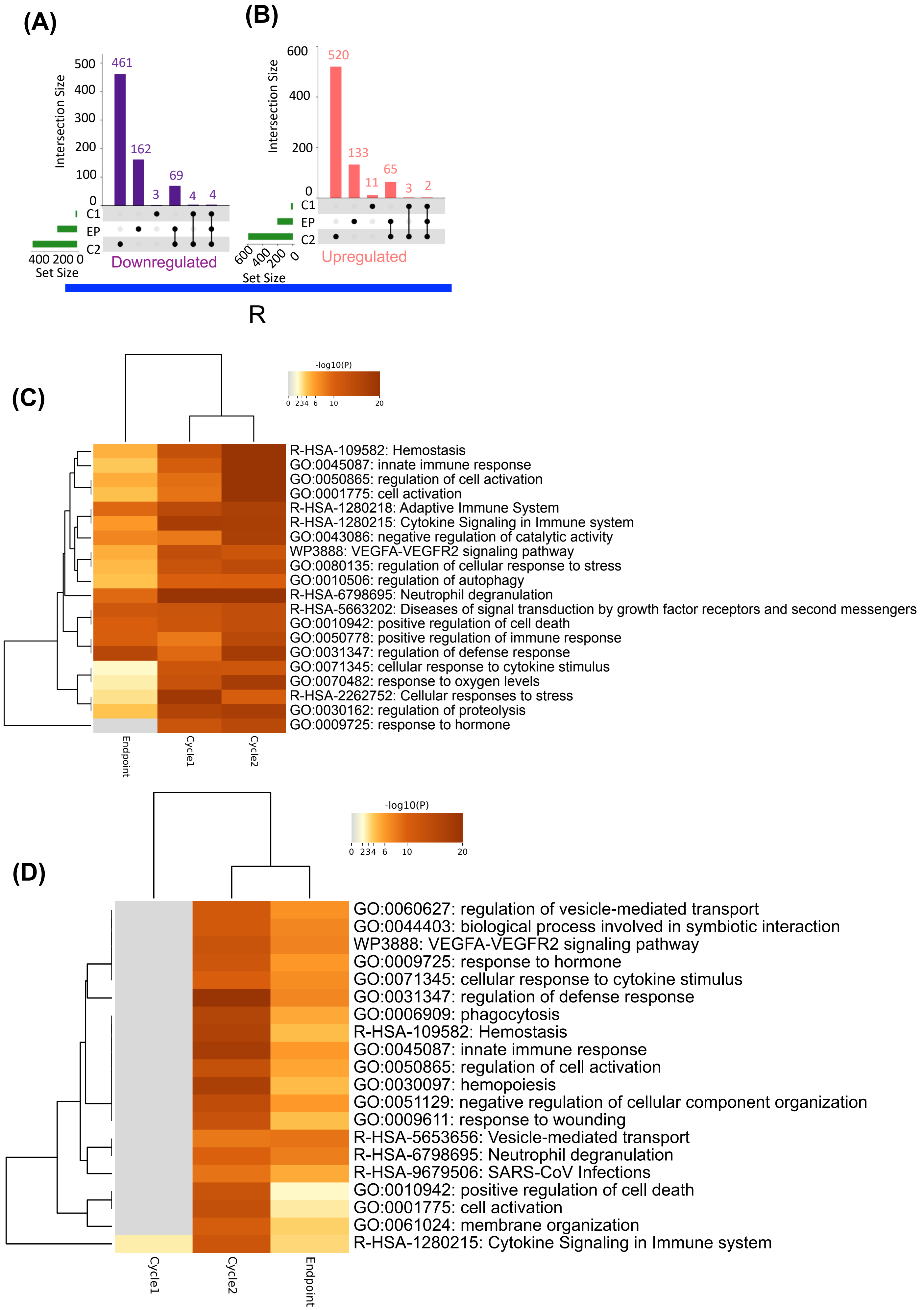
(A)
(B)
Intersection Size
461
162
3
69
4
4
C1
EP
C2
Set Size
Downregulated
520
133
11
65
3
2
Upregulated
R
(C)
-log10(P)
R-HSA-109582: Hemostasis
GO:0045087: innate immune response
GO:0050865: regulation of cell activation
GO:0001775: cell activation
R-HSA-1280218: Adaptive Immune System
R-HSA-1280215: Cytokine Signaling in Immune system
GO:0043086: negative regulation of catalytic activity
WP3888: VEGFA-VEGFR2 signaling pathway
GO:0080135: regulation of cellular response to stress
GO:0010506: regulation of autophagy
R-HSA-6798695: Neutrophil degranulation
R-HSA-5663202: Diseases of signal transduction by growth factor receptors and second messengers
GO:0010942: positive regulation of cell death
GO:0050778: positive regulation of immune response
GO:0031347: regulation of defense response
GO:0071345: cellular response to cytokine stimulus
GO:0070482: response to oxygen levels
R-HSA-2262752: Cellular responses to stress
GO:0030162: regulation of proteolysis
GO:0009725: response to hormone
Endpoint
Cycle1
Cycle2
(D)
-log10(P)
GO:0060627: regulation of vesicle-mediated transport
GO:0044403: biological process involved in symbiotic interaction
WP3888: VEGFA-VEGFR2 signaling pathway
GO:0009725: response to hormone
GO:0071345: cellular response to cytokine stimulus
GO:0031347: regulation of defense response
GO:0006909: phagocytosis
R-HSA-109582: Hemostasis
GO:0045087: innate immune response
GO:0050865: regulation of cell activation
GO:0030097: hemopoiesis
GO:0051129: negative regulation of cellular component organization
GO:0009611: response to wounding
R-HSA-5653656: Vesicle-mediated transport
R-HSA-6798695: Neutrophil degranulation
R-HSA-9679506: SARS-CoV Infections
GO:0010942: positive regulation of cell death
GO:0001775: cell activation
GO:0061024: membrane organization
R-HSA-1280215: Cytokine Signaling in Immune system
Cycle1
Cycle2
Endpoint

S Fig. 5

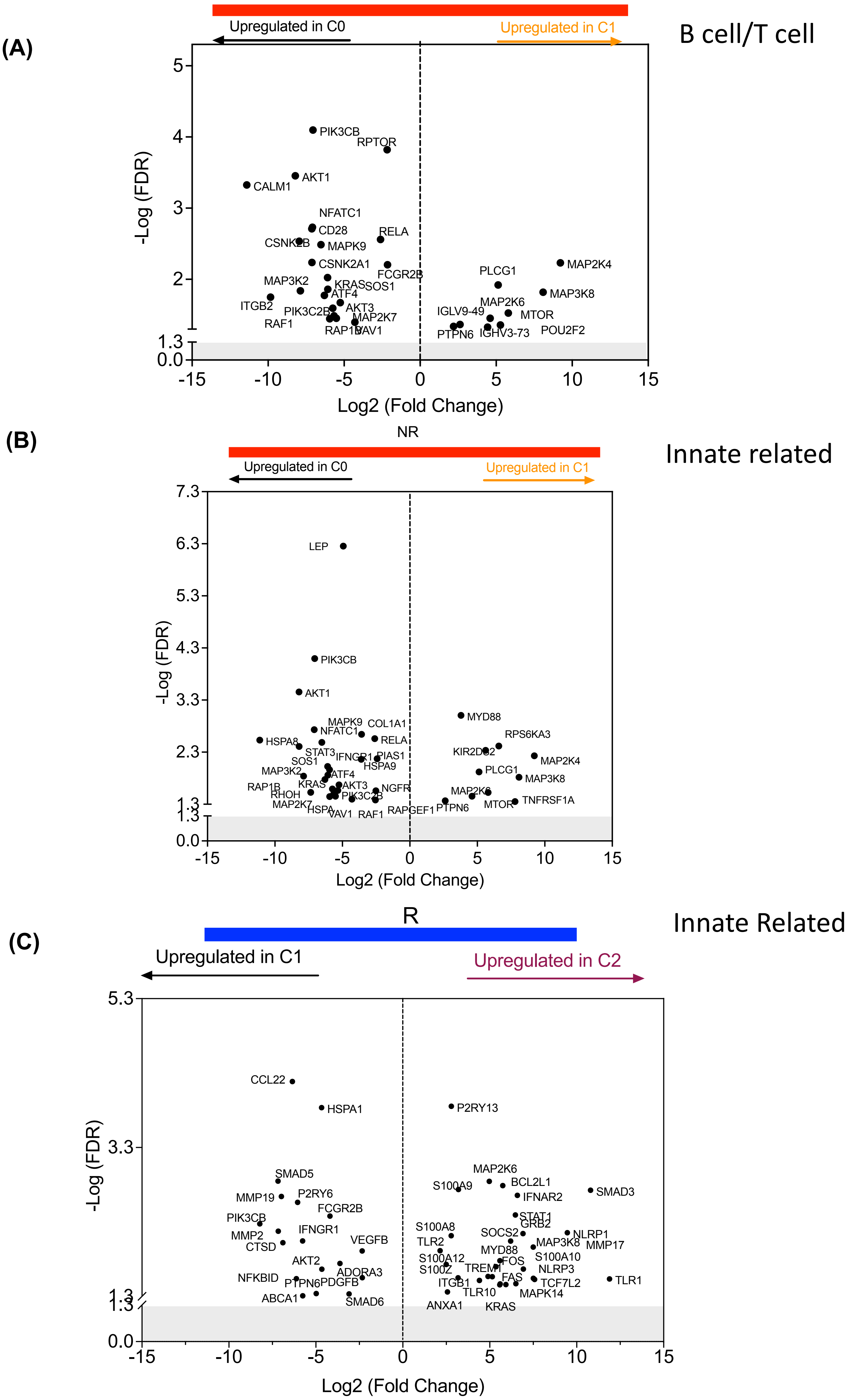
(A)
NR
Upregulated in C0
Upregulated in C1
B cell/T cell
PIK3CB
RPTOR
AKT1
CALM1
NFATC1
CD28
RELA
CSNK2B
MAPK9
CSNK2A1
FCGR2B
MAP3K2
KRAS
SOS1
ATF4
ITGB2
PIK3C2B
AKT3
MAP2K7
RAF1
RAP1B
VAV1
PLCG1
MAP2K4
MAP3K8
MAP2K6
IGLV9-49
MTOR
PTPN6
IGHV3-73
POU2F2
-Log (FDR)
Log2 (Fold Change)
(B)
NR
Upregulated in C0
Upregulated in C1
Innate related
LEP
PIK3CB
AKT1
MAPK9
NFATC1
COL1A1
HSPA8
RELA
STAT3
IFNGR1
PIAS1
SOS1
HSPA9
MAP3K2
ATF4
RAP1B
KRAS
AKT3
NGFR
RHOH
PIK3C2B
MAP2K7
HSPA
VAV1
RAF1
RAPGEF1
PTPN6
MYD88
RPS6KA3
KIR2DS2
MAP2K4
PLCG1
MAP3K8
MAP2K6
MTOR
TNFRSF1A
-Log (FDR)
Log2 (Fold Change)
(C)
R
Upregulated in C1
Upregulated in C2
Innate Related
CCL22
HSPA1
P2RY13
SMAD5
MMP19
P2RY6
FCGR2B
PIK3CB
MMP2
CTSD
IFNGR1
VEGFB
AKT2
ADORA3
NFKBID
PTPN6
PDGFB
ABCA1
SMAD6
MAP2K6
S100A9
BCL2L1
IFNAR2
SMAD3
STAT1
GRB2
S100A8
SOCS2
NLRP1
TLR2
MAP3K8
MMP17
MYD88
S100A12
FOS
S100A10
S100Z
TREM1
NLRP3
ITGB1
FAS
TCF7L2
TLR1
TLR10
MAPK14
ANXA1
KRAS
-Log (FDR)
Log2 (Fold Change)

S Fig. 6

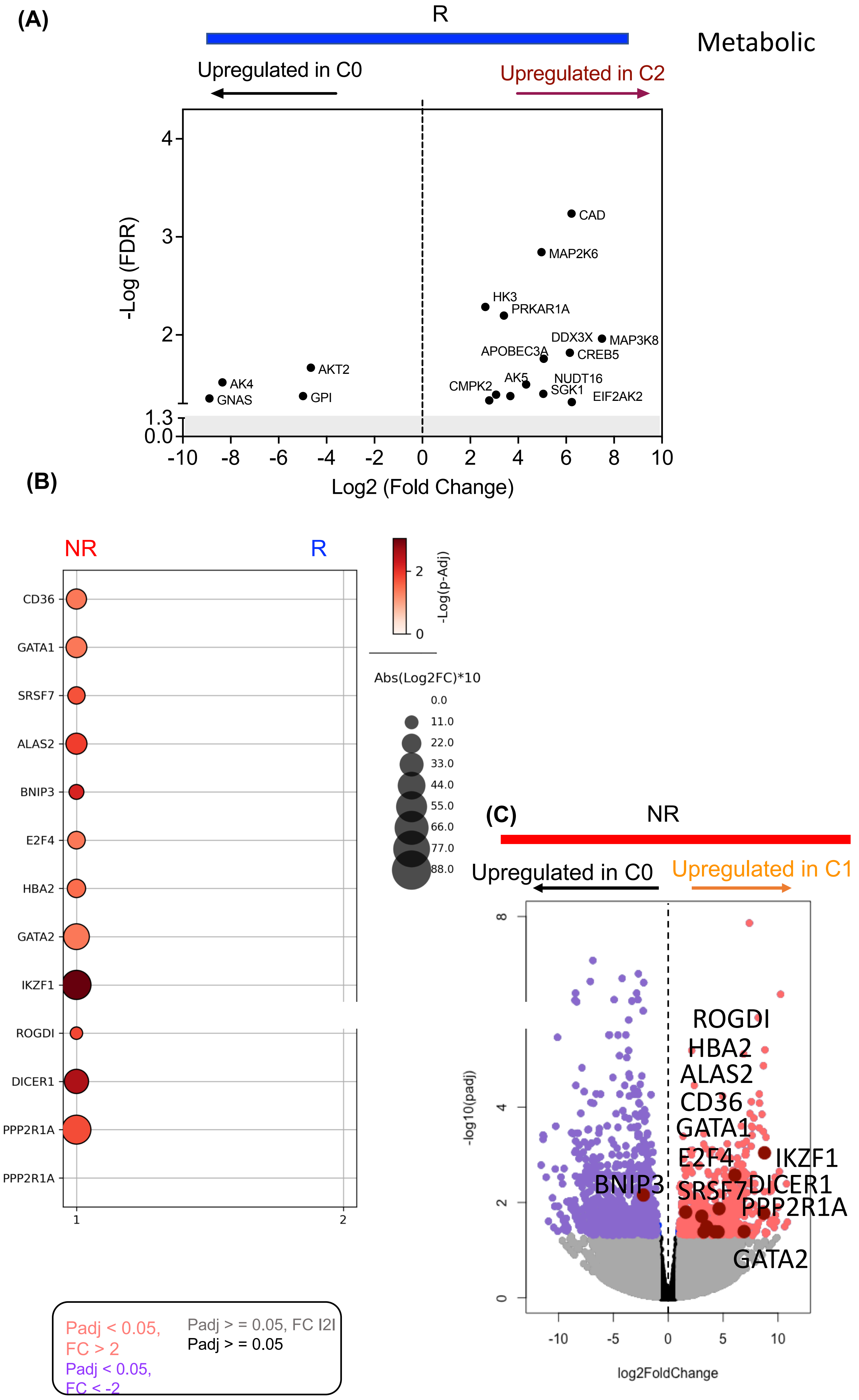
(A)
R
Metabolic
Upregulated in C0
Upregulated in C2
-Log (FDR)
Log2 (Fold Change)
CAD
MAP2K6
HK3
PRKAR1A
DDX3X
MAP3K8
APOBEC3A
CREB5
AKT2
AK4
GNAS
GPI
CMPK2
AK5
NUDT16
SGK1
EIF2AK2
(B)
NR
R
CD36
GATA1
SRSF7
ALAS2
BNIP3
E2F4
HBA2
GATA2
IKZF1
ROGDI
DICER1
PPP2R1A
PPP2R1A
-Log(p-Adj)
Abs(Log2FC)*10
(C)
NR
Upregulated in C0
Upregulated in C1
-log10(padj)
log2FoldChange
ROGDI
HBA2
ALAS2
CD36
GATA1
E2F4
IKZF1
BNIP3
SRSF7
DICER1
PPP2R1A
GATA2
Padj < 0.05, FC > 2
Padj < 0.05, FC < -2
Padj > = 0.05, FC I2I
Padj > = 0.05

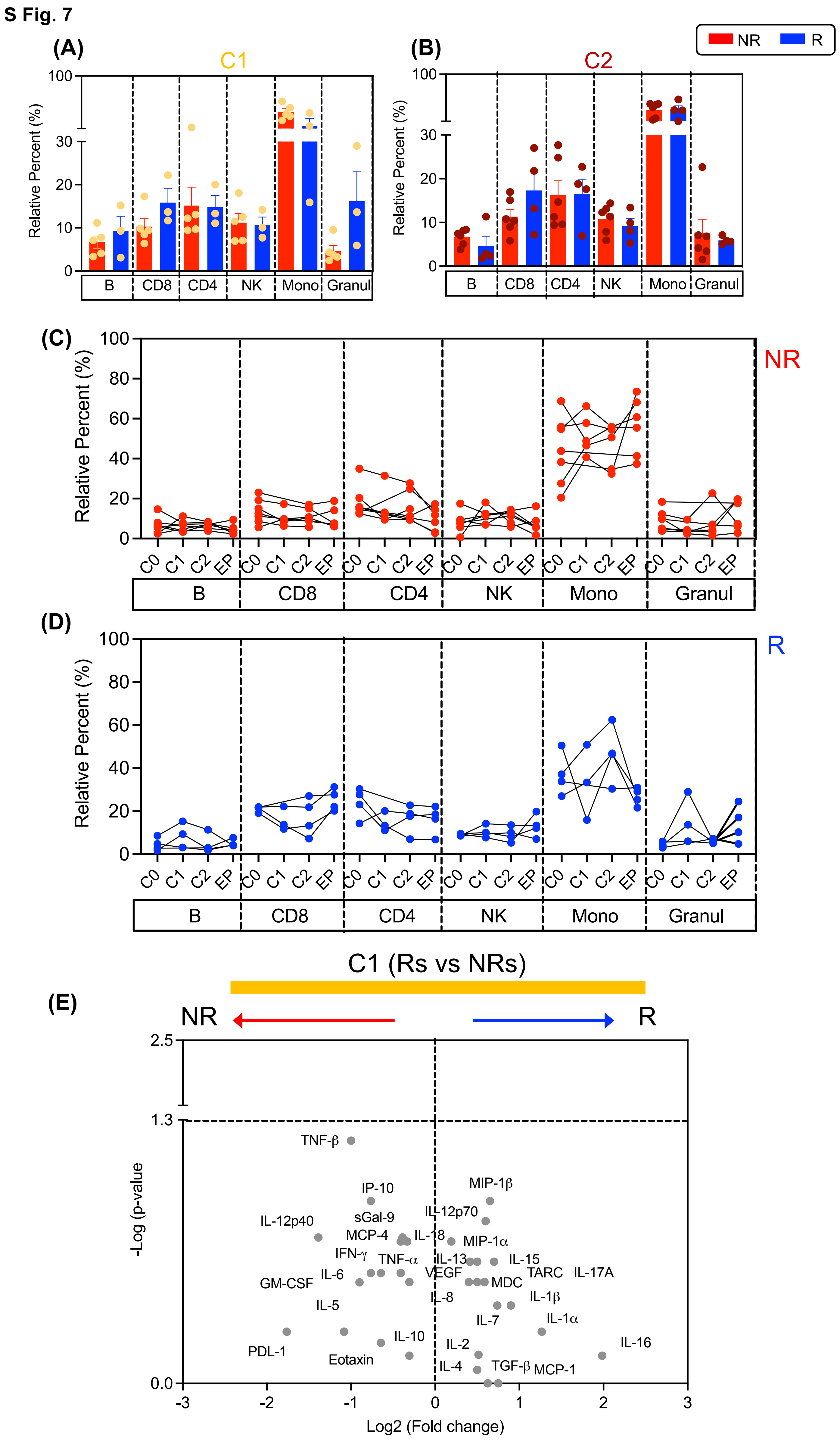
S Fig. 7
(A)
C1
(B)
C2
NR
R
Relative Percent (%)
B
CD8
CD4
NK
Mono
Granul
(C)
(D)
C0
C1
C2
EP
(E)
C1 (Rs vs NRs)
-Log (p-value
Log2 (Fold change)
TNF-β
IP-10
MIP-1β
IL-12p40
sGal-9
IL-12p70
MCP-4
IL-18
MIP-1α
IFN-γ
TNF-α
IL-13
IL-15
GM-CSF
IL-6
VEGF
TARC
IL-17A
MDC
IL-1β
IL-5
IL-8
IL-7
IL-1α
IL-10
IL-2
IL-16
PDL-1
Eotaxin
IL-4
TGF-β
MCP-1

S Fig. 8

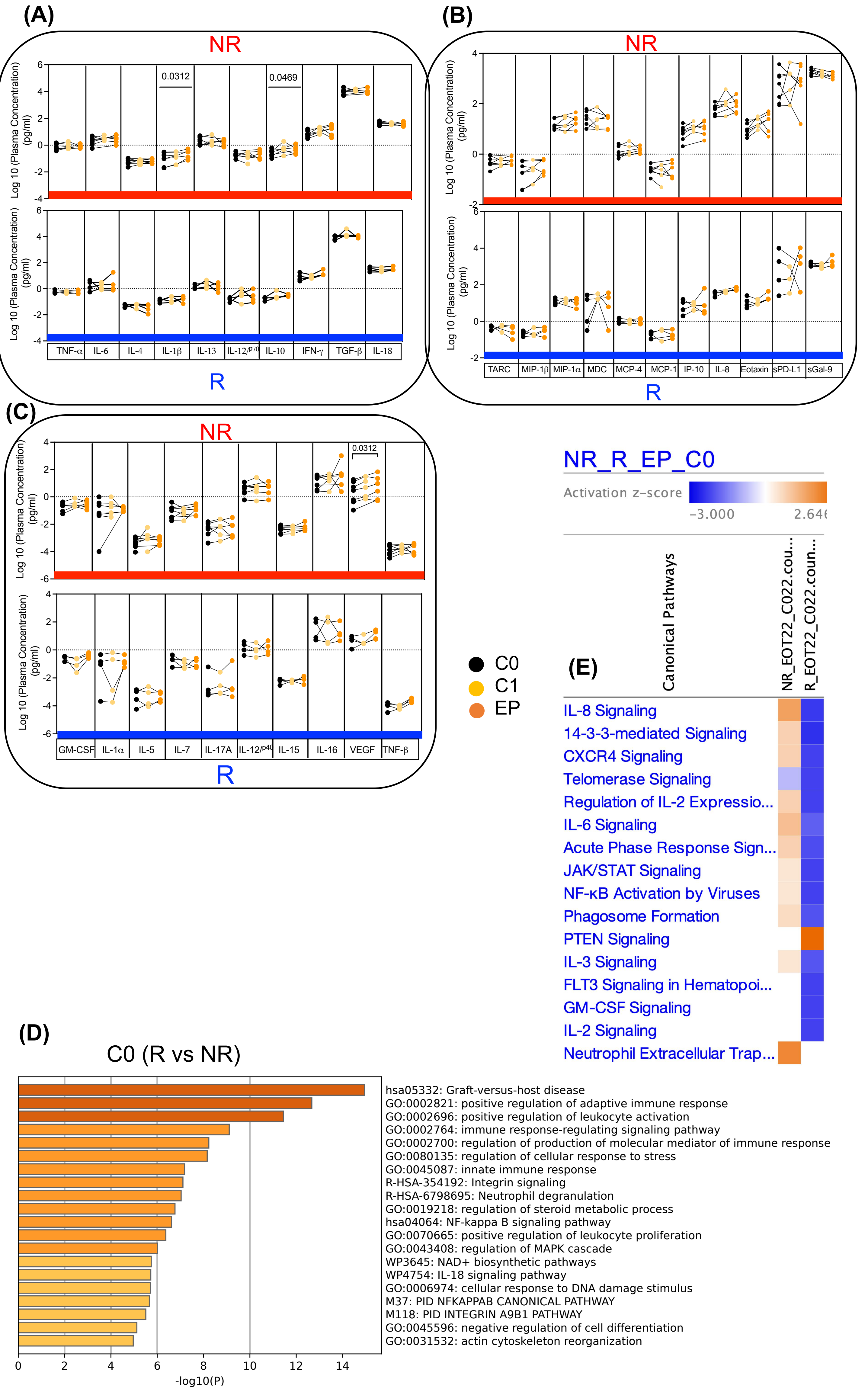

(A)
NR
R
Log 10 (Plasma Concentration) (pg/ml)
0.0312
0.0469
TNF-α
IL-6
IL-4
IL-1β
IL-13
IL-12/p70
IL-10
IFN-γ
TGF-β
IL-18
(B)
NR
R
TARC
MIP-1β
MIP-1α
MDC
MCP-4
MCP-1
IP-10
IL-8
Eotaxin
sPD-L1
sGal-9
(C)
NR
R
0.0312
GM-CSF
IL-1α
IL-5
IL-7
IL-17A
IL-12/p40
IL-15
IL-16
VEGF
TNF-β
C0
C1
EP
(D)
C0 (R vs NR)
hsa05332: Graft-versus-host disease
GO:0002821: positive regulation of adaptive immune response
GO:0002696: positive regulation of leukocyte activation
GO:0002764: immune response-regulating signaling pathway
GO:0002700: regulation of production of molecular mediator of immune response
GO:0080135: regulation of cellular response to stress
GO:0045087: innate immune response
R-HSA-354192: Integrin signaling
R-HSA-6798695: Neutrophil degranulation
GO:0019218: regulation of steroid metabolic process
hsa04064: NF-kappa B signaling pathway
GO:0070665: positive regulation of leukocyte proliferation
GO:0043408: regulation of MAPK cascade
WP3645: NAD+ biosynthetic pathways
WP4754: IL-18 signaling pathway
GO:0006974: cellular response to DNA damage stimulus
M37: PID NFKAPPAB CANONICAL PATHWAY
M118: PID INTEGRIN A9B1 PATHWAY
GO:0045596: negative regulation of cell differentiation
GO:0031532: actin cytoskeleton reorganization
-log10(P)
(E)
NR_R_EP_C0
Activation z-score
-3.000
2.646
Canonical Pathways
NR_EOT22_C022.cou...
R_EOT22_C022.coun...
IL-8 Signaling
14-3-3-mediated Signaling
CXCR4 Signaling
Telomerase Signaling
Regulation of IL-2 Expressio...
IL-6 Signaling
Acute Phase Response Sign...
JAK/STAT Signaling
NF-κB Activation by Viruses
Phagosome Formation
PTEN Signaling
IL-3 Signaling
FLT3 Signaling in Hematopoi...
GM-CSF Signaling
IL-2 Signaling
Neutrophil Extracellular Trap...

S Fig. 9

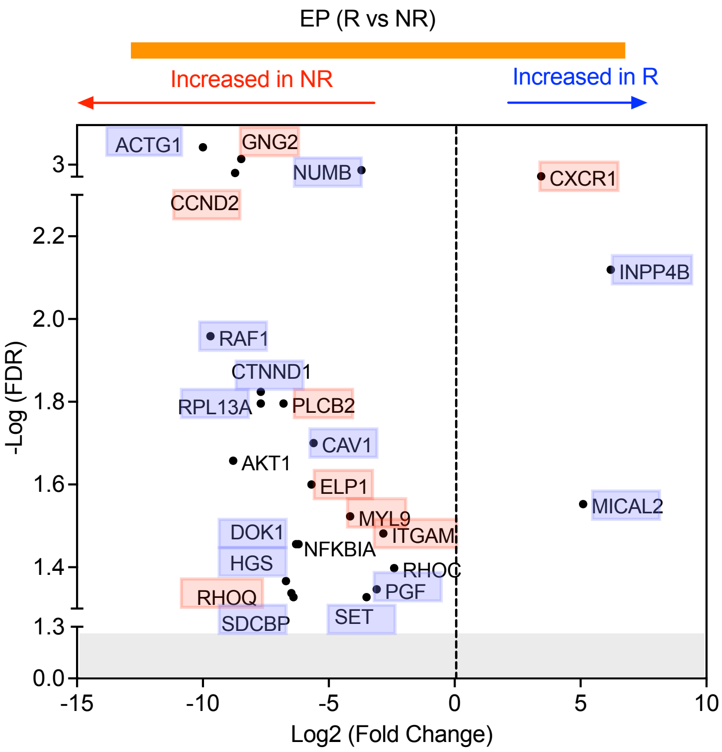
EP (R vs NR)
Increased in NR
Increased in R
-Log (FDR)
Log2 (Fold Change)
3
2.2
2.0
1.8
1.6
1.4
1.3
0.0
-15
-10
-5
0
5
10
ACTG1
GNG2
NUMB
CCND2
CXCR1
INPP4B
RAF1
CTNND1
RPL13A
PLCB2
CAV1
AKT1
ELP1
MICAL2
MYL9
ITGAM
DOK1
NFKBIA
HGS
RHOC
PGF
RHOQ
SDCBP
SET
