# Supplementary material for: Transcriptomic profiling of peripheral blood cells in HPV‐associated carcinoma patients receiving combined valproic acid and avelumab
Source: Mol Oncol. 2023 Sep 17;18(5):1209–30. doi: 10.1002/1878-0261.13519 (PMC11077001; doi:10.1002/1878-0261.13519)
Supplement: Supplementary file 2 — Table S1. Characteristics of samples used for RNA‐Seq. [file MOL2-18-1209-s004.docx]

**Supplementary Table 1.** Characteristics of samples used for RNAseq.

| **# Clinical Trial** | **# Sample** | **# Response Category** | **Cycle of Treatment** | **RIN** |
| --- | --- | --- | --- | --- |
| CCI-19 | NB1 | R2 | C0 | 7.5 |
| CCI-19 | NB2 | R2 | C2 | 9.2 |
| CCI-19 | NB3 | R2 | EP | 8.6 |
| CCI-20 | NB5 | NR4 | C0 | 7.4 |
| CCI-20 | NB6 | NR4 | C1 | 8.4 |
| CCI-20 | NB7 | NR4 | C2 | 8.8 |
| CCI-20 | NB8 | NR4 | EP | 9.5 |
| CCI-27 | NB18 | R4 | C0 | 6.8 |
| CCI-27 | NB19 | R4 | C1 | 9.4 |
| CCI-27 | NB20 | R4 | C2 | 9.5 |
| CCI-27 | NB21 | R4 | EP | 6.5 |
| CCI-29 | NB23 | NR2 | C0 | 8.6 |
| CCI-29 | NB24 | NR2 | C1 | 9.8 |
| CCI-29 | NB25 | NR2 | C2 | 9.2 |
| CCI-29 | NB26 | NR2 | EP | 8.6 |
| CCI-30 | NB27 | NR3 | C0 | 9.3 |
| CCI-30 | NB28 | NR3 | C1 | 8.5 |
| CCI-30 | NB29 | NR3 | C2 | 9.3 |
| CCI-31 | NB30 | R3 | C0 | 7.5 |
| CCI-31 | NB31 | R3 | C1 | 9.5 |
| CCI-31 | NB32 | R3 | C2 | 7.7 |
| CCI-31 | NB33 | R3 | EP | 7.6 |
| CCI-33 | NB35 | R1 | C0 | 7.1 |
| CCI-33 | NB36 | R1 | C1 | 8.9 |
| CCI-33 | NB37 | R1 | C2 | 8.9 |
| CCI-33 | NB38 | R1 | EP | 8.9 |
| CCI-38 | NB40 | NR5 | C0 | 8.8 |
| CCI-38 | NB41 | NR5 | C1 | 9.2 |
| CCI-38 | NB42 | NR5 | C2 | 7.1 |
| CCI-38 | NB43 | NR5 | EP | 8.5 |
| CCI-40 | NB44 | NR7 | C0 | 8.4 |
| CCI-40 | NB45 | NR7 | C1 | 9.5 |
| CCI-40 | NB46 | NR7 | C2 | 9.6 |
| CCI-40 | NB47 | NR7 | EP | 9.6 |
| CCI-43 | NB52 | NR6 | C0 | 7.3 |
| CCI-43 | NB54 | NR6 | EP | 6.6 |
| CCI-17 | NB55 | NR1 | C0 | 7.8 |
| CCI-17 | NB57 | NR1 | C2 | 7 |
| CCI-17 | NB58 | NR1 | EP | 8.4 |

R: Responders, NR: Non-responders, C0: Cycle 0, C1: Cycle 1, C2: Cycle 2, EP: Endpoint, RIN: RNA Integrity Number.
