# Supplementary material for: Transcriptomic profiling of peripheral blood cells in HPV‐associated carcinoma patients receiving combined valproic acid and avelumab
Source: Mol Oncol. 2023 Sep 17;18(5):1209–30. doi: 10.1002/1878-0261.13519 (PMC11077001; doi:10.1002/1878-0261.13519)
Supplement: Supplementary file 3 — Table S2. HPV‐associated metastatic patients' characteristics. [file MOL2-18-1209-s002.docx]

**Supplementary Table 2.** HPV-associated metastatic carcinoma patients’ characteristics (Ancillary cohort).

| **Response**  **Category** | **Tumor Site** | **Sex** | **Stage** | **Best Response** |
| --- | --- | --- | --- | --- |
| NR | Penis | M | IV | iUPD |
| R | Larynx | M | IVC | iCCR |
| R | Penis | M | IV | iCPR |
| R | Penis | M | IV | iUPR |
| R | Cervix | F | IVB | iSD |
| NR | Tonsil | M | IVC | iUPD |
| R | Nasopharynx | M | III | iUPR |
| NR | Tonsil | M | IV | iUPD |
| NR | Cervix | F | IVB | iCPD |
| NR | Anal | F | IV | iUPD |

R: Responders (iCCR+ iCPR+iUPR+ iSD), NR: Non-responders (iCPD+ iUPD+ Clinically progressive), M: Male, F: Female, iUPD: Unconfirmed progressive disease, iCCR: Confirmed complete response, iCPD: Confirmed progressive disease, NE: Response was not evaluated, iUPR: Unconfirmed partial response, iCPR: Confirmed partial response, iSD: Stable disease.
